# Treatment optimisation for blood pressure with single-pill combinations in India (TOPSPIN) – Protocol design and baseline characteristics

**DOI:** 10.1016/j.ijcrp.2024.200346

**Published:** 2024-10-24

**Authors:** Gaia Kiru, Ambuj Roy, Dimple Kondal, Ambalam M. Chandrasekaran, Somnath Mukherjee, Bishav Mohan, Kavita Singh, Hyndavi Salwa, Edmin Christa, Ameeka Shereen Lobo, Gayatri Mahajan, Aman Khanna, Amit Malviya, Satish G. Patil, Vinod K. Abichandani, Bhupinder Singh, Bal Kishan Gupta, Balsubramaiam Yellapantula, Dandge Shailendra, Shantanu Sengupta, Sunil Kumar, Neil Bardoloi, Mallika Khanna, Animesh Mishra, Kiran Aithal, Vipul Chavda, Victoria R. Cornelius, Dorairaj Prabhakaran, Neil Poulter, TOPSPIN Investigators

**Affiliations:** a Imperial Clinical Trials Unit, Imperial College London, 1st Floor, Stadium House, 68 Wood Lane, London, W12 7RH, UK, United Kingdom; b Department of Cardiology, All India Institute of Medical Sciences, Ansari Nagar, New Delhi, India; c Centre for Chronic Disease Control, Safdarjung Development Area, New Delhi, India; d DMC Heart Institute, Tagore Nagar, Civil Lines, Ludhiana, Punjab, India; e Heidelberg University, Heidelberg, Germany; f Aman Hospital & Research Center, Gotri Road, Vadodara, Gujarat, India; g North Eastern Indira Gandhi Regional Institute of Medical Sciences, Mawdiangdiang, East Khasi Hills, Shillong, Meghalaya, India; h Shri Dharmasthala Manjunatheshwara University, Manjushree Nagar, Sattur, Dharwad, Karnataka, India; i Rudraksh Hospital, Rudraksha Speciality Hospital, 1st Floor Raj Mandir Complex, Bareja, Ahmedabad, India; j Department of Cardiology All India Institute of Medical Sciences, Bhatinda, Punjab, India; k Department of Medicine, S.P. Medical College & A.G. of Hospitals Bikaner, Rajasthan, India; l Shalinitai Meghe Hospital and Research Center, Higna Road, Wandongari, Nagpur, Maharashtra, India; m Mediciti Institute of Medical Sciences, Hyderabad, India; n Sengupta Hospital & Research Institute, Ravinagar Square, Nagpur, 440033, Maharashtra, India; o JSS Hospital Mahathma Gandhi Road, Fort Mohalla, Mysuru, India; p Apollo Excelcare Hospital, Paschim Boragaon, Guwahati, Assam, 781033, India

## Abstract

**Background:**

The burden of over 300 million individuals living with hypertension in India is increasing steadily. Most current guidelines recommend initial combination therapy for effective blood pressure (BP) control. However, there is no randomised evidence to inform which combinations to use in the South Asian population, who account for over one-quarter of the world's population.

**Methods:**

This multi-centre, single-blind, randomised, three-arm trial recruited men and women aged 30–79 years with hypertension. The trial compares the efficacy of commonly recommended single pill combinations (SPCs) of three drug classes – calcium channel blocker (amlodipine), ACE inhibitor (perindopril), and a thiazide-like diuretic (indapamide). The primary objective is to determine the most effective two-drug combination, initially at starting doses with forced up-titration at 2 months, in reducing 24-h ambulatory systolic blood pressure (ASBP) at 6 months. The trial has 85 % power to detect a difference of 3 mmHg in 24-h ASBP amongst the groups.

Participant recruitment took place from August 2022 to February 2024.

**Baseline results:**

The 1981 participants (42.0 % women) enrolled had a mean age of 52.1 (SD 11.3) years and a mean body mass index of 26.5 (SD 4.2) kg/m2. 58.1 % of participants had a previous diagnosis of hypertension and 18.6 % of participants were known to diabetes. The mean ASBP was 135.6 (SD 17.0) mmHg, and the mean ambulatory diastolic BP was 84.5 (SD 10.9) mmHg.

**Conclusion:**

The TOPSPIN trial is the first randomised evaluation of commonly used BP-lowering combination therapies in a South Asian population. The results have potentially significant implications for choosing first-line antihypertensive agents among Indians and the South Asian diaspora.

## Highlights

• Guidelines recommend combination therapy as initial hypertension treatment, ideally in a single pill.
• Hitherto, no randomised data on choice of 2-drug combinations exist for South Asians.
• TOPSPIN compares BP-lowering efficacy of 3 commonly used single pill combinations of 2 anti-hypertensive agents.
• TOPSPIN may have implications for better BP control in India and the South Asian diaspora.

## 1 Introduction

Hypertension is a significant global health concern, affecting approximately 25 % of adults, with only a minority of those achieving even conservative blood pressure (BP) targets [[1], [2], [3]]. Elevated systolic blood pressure (SBP) is recognised as the leading risk factor for morbidity and mortality worldwide [2,3]. In India, hypertension has an adult prevalence of about 30 % (28 % in rural and 34 % in urban populations) and is a leading cause of death and disability [[4], [5], [6]]. Furthermore, hypertension control rates in India are even lower, at 8–11 % in rural areas and 8–20 % in urban [[6], [7], [8]].

Achieving target BP levels is crucial in mitigating cardiovascular events among hypertensive patients. According to recent guidelines, this typically necessitates using at least two BP-lowering medications, as monotherapy effectively achieves optimal BP control for only approximately 30 % of patients [9,10]. Current recommendations advocate the initiation of combination therapy as a first-line treatment in most adults and particularly in patients with comorbid conditions that necessitate rapid BP reduction [10,11]. These combinations usually include an angiotensin-converting enzyme (ACE) inhibitor, or an angiotensin II receptor blocker (ARB) paired with a calcium channel blocker (CCB) or a thiazide/thiazide-like diuretic [9]. Similarly, the latest Indian hypertension guidelines endorse the use of low doses of drug combinations to achieve the desired BP control [11].

Multiple randomised controlled trials have demonstrated that reducing BP is associated with decreased cardiovascular morbidity and mortality [[12], [13], [14], [15], [16]]. Randomised controlled trials are also consistent in showing that most patients with hypertension require at least two BP lowering agents to reach recommended targets [[17], [18], [19], [20], [21], [22], [23], [24], [25], [26]]. Consequently, there has been increased emphasis in recent guidelines on using combinations of two or three agents to manage hypertension [10,27]. In addition, fixed-dose single-pill combinations (SPCs) of antihypertensive agents improve adherence and efficacy of BP control over monotherapies or two pill combinations [26,28]. This was the rationale for the design and conduct of the CREOLE trial, which compared three two-drug combinations of BP-lowering agents in black patients in Sub-Saharan Africa and preceded TOPSPIN [29].

No trials on the optimal initial antihypertensive combination have been conducted in India, nor have enough South Asian individuals been included in international trials. As a result, Indian guidelines on antihypertensive drug selection and sequencing are primarily extrapolated from international guidelines, which may or may not apply to the Indian patient population. The TOPSPIN trial addresses this critical knowledge gap by evaluating the efficacy of three commonly recommended two-drug combinations of antihypertensive therapies on 24-h ambulatory BP levels in Indian patients.

## 2 Design and Methods

### 2.1 Trial objectives and endpoints

The primary objective is to determine which of three SPCs of two anti-hypertensive agents (Amlodipine and Perindopril; Perindopril and Indapamide; Amlodipine and Indapamide) is most effective in reducing 24-h ambulatory systolic BP (ASBP) at 6 months adjusted for baseline ASBP (the difference between the mean ASBP at randomisation and that at 6 months) [30].

The trial's secondary objectives include evaluating the efficacy, safety and side effect profile of the three SPCs of two antihypertensive agents. These are

1. Reduction in 24-h ambulatory diastolic BP (ADBP) adjusted for baseline ADBP.
2. Reduction in daytime and night-time BP at six months adjusted for baseline values.
3. Reduction in clinic systolic and diastolic BP at two, four and six months adjusted for baseline values.
4. 24-hour and within-visit clinic BP variability.
5. Proportion of patients who achieve BP control (primarily defined as clinic BP < 140/90 mmHg) at two, four and six months and ABPM measured control (<130/80 mmHg) at six months. In addition, more contemporary guideline definitions of control of clinic BP will also be evaluated as BP < 130/80 mmHg.
6. Proportion of “responders” (defined as clinic BP reduction ≥20 mmHg SBP and ≥10 mmHg DBP) at any of the clinic visits (two, four and six months).
7. Changes in laboratory parameters such as micro- and macro-albuminuria, fasting blood glucose, lipid profiles, serum electrolytes, urea, creatinine and estimated glomerular filtration rate at six months adjusted for baseline values.
8. Adverse event (AE) rates in each arm leading to withdrawal from the trial.

To determine whether baseline plasma renin and aldosterone levels predict differential BP effects of the SPCs.

### 2.2 Trial population

The trial involves patients with essential hypertension from secondary and tertiary care clinics and hospitals across India. Patients from primary healthcare settings were also invited to participate and enrol at secondary or tertiary care trial recruiting hospitals.

Participants were required to meet the following criteria.

1. Age: 30–79 years.
2. Blood Pressure Criteria:
  o Clinic SBP ≥140 mmHg and <160 mmHg while on one antihypertensive agent or
  o Clinic SBP ≥150 mmHg and <180 mmHg with no antihypertensive treatment.
  o Measurements are based on the average of the last two of the three readings taken while seated in a clinic.

Participants were excluded if they had any of the following conditions.

1. Clinically defined congestive heart failure.
2. History of intolerance to any of the trial medications (e.g., angioedema or dry cough with ACE inhibitors).
3. Serum creatinine >132.6 μmol/L (1.5 mg/dL).
4. History of coronary heart disease (e.g., chronic stable angina, myocardial infarction, or acute coronary syndrome).
5. History of stroke or other cerebrovascular events (e.g., transient ischemic attack or reversible ischemic neurological deficit).
6. Severe hepatic impairment.
7. Concurrent treatment with agents known to cause torsade's de pointes.
8. Current lactation.
9. Contraindications to any trial drugs per their Summary of Product Characteristics (SmPC).
10. Known or suspected secondary hypertension.
11. Any other illness or impairment that could interfere with the trial conduct.
12. Pregnancy or women of childbearing potential not using reliable contraception.
13. History of gout.
14. Serum potassium <3.5 mmol/L at screening.

### 2.3 Design

TOPSPIN is a randomised, single-blind, multi-centre, three-arm trial to assess the efficacy of different antihypertensive drug combinations in patients of South Asian origin. The trial recruited hypertensive patients from outpatient clinics at 35 hospitals across India from August 2022 to February 2024. The principal investigators or an assignee informed eligible patients about the study's purpose, procedures, risks, and benefits. After obtaining written informed consent, investigations were undertaken as per Table 1. All data were collected using the CLINION database,a validated electronic clinical data management system hosted by the Public Health Foundation of India. Any AEs that cause drug discontinuation were recorded at every visit, and all/any serious adverse events (SAEs) are recorded at clinic visits in compliance with the New Drugs and Clinical Trials Rules, 2019 and The Pharmaco-vigilance Programme of India (PvPI) as per local requirements. Before the commencement of the trial, all regulatory and ethical approvals were obtained at the coordinating centre and each centre.

**Table 1:** TOPSPIN investigations.

Investigations	Visit 0: Screening	Visit 1: Randomisation	Visit 2: 2 mths^a^	Visit 3: 4 mths^a^	Visit 4: 6 mths^a^
Informed Consent	×	–	–	–	–
Medical and Social History, including major changes to smoking, diet and alcohol consumption	×	×	×	×	×
Height	–	×	–	–	–
Weight	×	×	×	×	×
Pulse	×	×	×	×	×
Check for Renal Bruit and Radio Femoral Delay	×	–	–	–	–
24h-Ambulatory BP Measurement^b^	–	×	–	–	×
Clinic BP Measurement^c^	×	×	×	×	×
Phlebotomy (fasting blood glucose and lipid profile, urea, uric acid, creatinine, eGFR, K+, Na+) ± serum/plasma for storage	×	–	–	–	×
Urine collection for micro-albuminuria and proteinuria	×	–	–	–	×
Trial drug dispensing	–	×	×	×	–
Trial drug accountability	–	–	×	×	×
Adverse Events and Serious Adverse Events	–	–	×	×	×

a visit window period at 2 month, 4month and 6 months will be ± 10 days around the actual visit date.

b Patients wear the ABPM device for a minimum of 24 h with automatic readings every 30 min both in the daytime and at nighttime. Daytime is defined as 9 a.m. through 9 p.m., and night-time as 12 midnight through 6 a.m. Small, medium or large BP cuffs are utilised as appropriate on the non-dominant arm. ABPM recordings will only be accepted if at least 70 % of expected readings are included. ABPM recordings which do not meet this requirement will need to be repeated.

c average of the second and third readings.

### 2.4 Trial treatment

Participants were randomised in a 1:1:1 ratio to one of the three treatment arms as in Table 2. Indapamide SR is only available at a dose of 1.5 mg in an SPC with amlodipine. This is the maximal dose of Indapamide SR. Hence, in the interest of using only SPCs, for the first 2 months, there was a degree of imbalance across the 3 groups – specifically that the diuretic in the amlodipine-indapamide arm was not initiated at a half dose like the other components of the 3 SPCs. However, the BP lowering efficacy of the indapamide SR 1.5 mg formulation is equivalent to the indapamide 2.5 mg formulation combined with perindopril. Therefore, between months two and six, all medicines in the SPCs were at the usual maximum clinical dosage.

**Table 2:** Summary of trial treatment regimens.

Treatment Arm	Treatment Period 1: Enrolment – 2 months	Treatment Period 2: 2 months–6 months (if SBP ≥120 mmHg)
1	Amlodipine 5 mg + Perindopril 4 mg	Amlodipine 10 mg + Perindopril 8 mg
2	Perindopril 4 mg + Indapamide 1.25 mg	Perindopril 8 mg + Indapamide 2.5 mg
3	Amlodipine 5 mg + Indapamide 1.5 mg SR^a^	Amlodipine 10 mg + Indapamide 1.5 mg SR^a^

a sustained release.

Using a blocked design, randomisation was stratified by age (<55 or ≥55 years) and trial site. After two months, if a participant's SBP remains ≥120 mmHg, their medication dose was increased, and they continued this regimen for the trial's duration. If, after a further two months, clinic SBP exceeded 160 mmHg or diastolic BP (DBP) exceeded 100 mmHg, a beta-blocker (bisoprolol 5 mg) was added to the treatment regimen unless contraindicated. In cases where a beta-blocker was not suitable, an alternative agent that is not a thiazide or thiazide-like diuretic, ACE inhibitor, angiotensin receptor blockers or CCB, such as spironolactone 12.5 mg or doxazosin 2 mg could be added at the investigator's discretion. Tablet counts are performed at each visit (two, four, and six months) to assess adherence, defined as taking ≥80 % of the prescribed doses. Adherence in the 24-h prior to each visit is documented.

## 3 Statistical analyses

### 3.1 Sample size and power Considerations

To detect a clinically meaningful difference of 3.0 mmHg between arms in the 24-h mean ASBP assuming an SD in 24-h ASBP of 15 mmHg with 85 % power and adjusting for three comparisons using a two-sided significance level of 0.0167, we needed a minimum of 590 participants per arm. Factoring in a 10 % dropout rate, we were required to recruit a total of 1968 participants (656 participants per arm) to achieve 590 evaluable participants per group.

### 3.2 Analysis of primary and secondary outcomes

The trial's data analysis, conducted by a blinded statistician, will adhere to CONSORT guidelines. Baseline participant data will be summarised by treatment arms without significance testing. The primary analysis will include all randomised participants, with multiple imputations for missing data. A linear model will compare ASBP differences between treatment arms, accounting for baseline ASBP, age, and recruitment site. Adjustments for multiple testing will use the Hommel method. Similar analyses will be conducted for secondary outcomes, with repeated measures analysed using linear mixed models and binary outcomes through logistic regression. Stata version 16.0 will be used for all analyses (See Statistical analysis plan in supplementary appendix) [[31], [32], [33], [34], [35], [36]].

## 4 Baseline results

The trial cohort consists of 1981 hypertensive participants with a mean age of 52.1 years (SD 11.3) and a median age of 52.0 years (IQR 44.0–60.0), of whom 42 % are women and 58 % are men (Table 3). Participants had a mean BMI of 26.5 (SD 4.2) kg/m2. At baseline, 58 % had a previous diagnosis of hypertension, while 42 % were newly diagnosed. Diabetes was present in 18.6 % of the cohort, and 7.3 % had dyslipidaemia. The prevalence of current smoking was low, at 6.2 %. Symptoms such as dyspnea (5.7 %), orthopnea (4.3 %), and palpitations (3.4 %) were uncommon. There was no history of coronary heart disease or stroke among participants.

**Table 3:** Baseline Demographics, Medical history and blood pressure (n = 1981).

Age (years), mean (SD)	52.1 (11.3)
≥55 years	798 (40.3 %)
<55 years	1183 (59.7 %)
Men	1147 (58.0 %)
Women	834 (42.0 %)
Weight (Kg), mean (SD)	69.4 (12.2)
Height (cm), mean (SD)	161.9 (9.4)
BMI (Kg/m^2^), mean (SD)	26.5 (4.2)
Previously Diagnosed Hypertension^a^	1149 (58.0 %)
Previously Diagnosed Diabetes^a^	369 (18.6 %)
Previously Diagnosed Dyslipidaemia^a^	144 (7.3 %)
Smoking	
Former	198 (10.0 %)
Current	123 (6.2 %)
Alcohol	
Former	153 (7.7 %)
Current	182 (9.2 %)
Average Clinic Systolic Blood Pressure (mmHg)	155.4 (9.7)^b^
Average Clinic Diastolic Blood Pressure (mmHg)	92.0 (10.8)^b^
Average Clinic Heart rate (beats/minute)	83.6 (13.1)^b^
Ambulatory Systolic Blood pressure (mmHg), mean (SD)	
24hr	135.6 (17.0)
Daytime	140.0 (17.1)
Nighttime	126.5 (20.5)
Ambulatory Diastolic Blood pressure (mmHg), mean (SD)	
24hr	84.5 (10.9)
Daytime	87.9 (11.3)
Nighttime	77.9 (12.9)

a Self-reported history.

b average of 2nd and 3rd readings.

The baseline mean clinic SBP was 155.4 mmHg (SD 9.7), and the mean clinic DBP was 92.0 mmHg (SD 10.8). The mean heart rate was 83.6 beats per minute (SD 13.1) (Table 3).

Mean 24-h ASBP was 135.6 mmHg (SD 17.0) with a median of 135.0 mmHg (IQR 125.0–147.0). Mean daytime ASBP was 140.1 mmHg (SD 17.1), while mean nighttime ASBP was 126.5 mmHg (SD 20.5). Mean 24-h ADBP was 84.5 mmHg (SD 10.9), and daytime and nighttime ADBP were 87.9 mmHg (SD 11.3) and 77.9 mmHg (SD 12.9), respectively (Table 3).

At baseline, 55.5 % of participants were on BP-lowering medication, while lipid-lowering and glucose-lowering medications were used by 9.1 % and 17.0 %, respectively (Table 4). Antiplatelet medication was reported by 2.3 % of participants, and the use of other medicines including vitamins/minerals/traditional/alternative/other was 11.3 % of the cohort.

**Table 4:** Baseline medication and non-pharmacological activities.

	N = 1981
**Self-reported medication**	**n (%)**
Blood pressure lowering	1100 (55.5 %)
Lipid-lowering	181 (9.1 %)
Glucose lowering	336 (17.0 %)
Antiplatelet	46 (2.3 %)
Other medications^a^	223 (11.3 %)
**Non-pharmacological approaches to BP reduction**	**n (%)**
Physical activity	1091 (55.1 %)
Salt restriction	993 (50.1 %)
Calorie restriction	626 (31.6 %)
Yoga	495 (25.0 %)
Dietary Supplements	246 (12.4 %)
Moderation of alcohol	202 (10.2 %)

a Other medications include vitamins/minerals/traditional/alternative/other medications.

Non-pharmacological activities related to BP management include physical activity, practised by 55.1 % of participants, and salt restriction, reported by 50.1 %. Other measures reported for BP reduction was calorie restriction reported by 31.6 %, yoga by 25.0 %, dietary supplements by 12.4 % and alcohol moderation by 10.4 % (Table 4).

At baseline, 10 % were former smokers, and 6.2 % reported current smoking, while 7.7 % and 9.2 % reported being former and current alcohol users, respectively (Table 3).

## 5 Discussion

Most current international guidelines on hypertension recommend drug-treatment initiation with two BP lowering agents (preferably as SPCs). However, no data are available to inform which combinations of BP-lowering agents are most effective in the Indian population. Hence, the TOPSPIN trial was designed to assess the efficacy and safety of three SPCs of antihypertensive therapies in the Indian population. The trial compares SPCs of amlodipine/perindopril, perindopril/indapamide, and amlodipine/indapamide, initially at starting doses with uptitration to full doses at two months.

The rationale for selecting these specific combinations lies in their established efficacy in reducing BP levels and major adverse cardiovascular events. Further, these three combinations of drug classes are the most commonly recommended in current guidelines. SPCs are preferred because they lower BP more rapidly and achieve better BP control than monotherapy, potentially reducing adverse effects associated with higher doses of individual agents. Furthermore, these combinations have demonstrated favourable safety profiles in previous clinical trials. However, due to the available range of doses used in the SPCs used in TOPSPIN, there was some dosing imbalance across the three arms during Period 1 (Table 2). Specifically in Period 1, amlodipine 5 mg is combined with indapamide sustained release (SR) 1.5 mg (the only available and full dose of indapamide SR). In contrast, perindopril is combined with indapamide immediate release (IR) at 1.25 (starting dose). However, between two and six months, full doses of all six components of the 3 SPCs were used for comparison (Table 2) (indapamide IR 2.5 mg and indapamide SR 1.5 mg produce equivalent BP-lowering) [37].

This trial employs a randomised, single-blind, multi-centre design to ensure robustness and minimise bias. Centralised randomisation and blinding procedures enhance the trial's internal validity, allowing for unbiased assessment of treatment outcomes. The inclusion criteria are designed to enrol a broad representative sample of hypertensive patients in India taking either none or one BP lowering agent (most hypertensive patients in India), thereby ensuring the generalisability of the results to this large population. The findings from this trial can potentially provide practice-changing inputs for the optimal management of hypertension among Indians. Understanding which combination therapy offers the best balance of efficacy and safety will guide clinicians in selecting appropriate treatment strategies. This trial will help guide hypertension management among the large population of South Asians living in the subcontinent and abroad, who comprise approximately one-quarter of the global population.

Challenges and limitations of the trial was timely recruitment, as patients of uncomplicated hypertension are typically not managed in secondary and tertiary care hospitals, where most research is conducted. The trial used marketed SPCs for logistical ease and thus not all possible anti-hypertensive combinations, such as one with a beta-blocker, were tested. The trial endpoints were reduction in ambulatory and clinic BP and not major adverse cardiovascular events due to funding constraints. Lastly, maintaining patient adherence and minimising loss to follow-up were the challenges.

## 6 Conclusion

This protocol paper outlines a trial which is the first to provide data on the BP-lowering efficacy and safety in the Indian population of the three most commonly recommended two-drug anti-hypertensive agents. It is sufficiently powered for results to be generalisable across a broad spectrum of Indian patients with 42 % female representation and a wide age range of 30–79 years. The findings may have significant clinical implications for improving BP control in India and the South Asian diaspora.

## CRediT authorship contribution statement

**Gaia Kiru:** Writing – original draft, Supervision, Project administration, Methodology, Data curation, Conceptualization. **Ambuj Roy:** Writing – review & editing, Writing – original draft, Visualization, Validation, Supervision, Resources, Project administration, Methodology, Investigation, Funding acquisition, Formal analysis, Data curation, Conceptualization. **Dimple Kondal:** Writing – review & editing, Writing – original draft, Visualization, Validation, Supervision, Methodology, Formal analysis, Data curation, Conceptualization. **Ambalam M. Chandrasekaran:** Writing – review & editing, Supervision, Project administration, Formal analysis, Data curation. **Somnath Mukherjee:** Writing – review & editing, Writing – original draft, Validation, Supervision, Software, Project administration, Formal analysis, Data curation. **Bishav Mohan:** Writing – review & editing, Investigation. **Kavita Singh:** Writing – review & editing, Validation, Supervision, Software, Project administration, Methodology, Data curation, Conceptualization. **Hyndavi Salwa:** Writing – review & editing, Validation, Supervision, Project administration, Data curation. **Edmin Christa:** Writing – review & editing, Validation, Supervision, Project administration, Data curation. **Ameeka Shereen Lobo:** Writing – review & editing, Visualization, Validation, Supervision, Project administration, Conceptualization. **Gayatri Mahajan:** Writing – review & editing, Validation, Supervision, Project administration, Data curation. **Aman Khanna:** Writing – review & editing, Investigation, Data curation. **Amit Malviya:** Writing – review & editing, Investigation, Data curation. **Satish G. Patil:** Writing – review & editing, Investigation, Data curation. **Vinod K. Abichandani:** Writing – review & editing, Investigation, Data curation. **Bhupinder Singh:** Writing – review & editing, Investigation, Data curation. **Bal Kishan Gupta:** Writing – review & editing, Investigation, Data curation. **Balsubramaiam Yellapantula:** Writing – review & editing, Investigation, Data curation. **Dandge Shailendra:** Writing – review & editing, Investigation, Conceptualization. **Shantanu Sengupta:** Writing – review & editing, Investigation, Data curation. **Sunil Kumar:** Writing – review & editing, Investigation, Data curation. **Neil Bardoloi:** Writing – review & editing, Investigation, Data curation. **Mallika Khanna:** Writing – review & editing, Investigation, Data curation. **Animesh Mishra:** Writing – review & editing, Investigation, Data curation. **Kiran Aithal:** Writing – review & editing, Investigation, Data curation. **Vipul Chavda:** Writing – review & editing, Investigation, Data curation. **Victoria R. Cornelius:** Writing – review & editing, Writing – original draft, Visualization, Validation, Methodology, Investigation, Formal analysis, Data curation, Conceptualization. **Dorairaj Prabhakaran:** Writing – review & editing, Writing – original draft, Visualization, Validation, Supervision, Resources, Project administration, Methodology, Investigation, Funding acquisition, Formal analysis, Data curation, Conceptualization. **Neil Poulter:** Writing – review & editing, Writing – original draft, Visualization, Validation, Supervision, Resources, Project administration, Methodology, Investigation, Funding acquisition, Formal analysis, Data curation, Conceptualization.

## Funding

This work was supported by Servier Affaires Médicales, France; Servier India Private Limited, India; 10.13039/501100000761Imperial College London, United Kingdom; and the Centre for Chronic Disease Control, India.

## Declaration of Competing interest

Neil Poulter has received financial support from several pharmaceutical companies which manufacture BP-lowering agents, for consultancy fees (Servier, Aktiia), research projects and staff (Servier, Pfizer) and for arranging and speaking at educational meetings (AstraZeneca, Lri Therapharma, Napi, Servier, Sanofi, Eva Pharma, Pfizer, Emcure India, Dr Reddy’s Laboratories and Zydus). He holds no stocks and shares in any such companies.
