# Supplementary material for: Treatment optimisation for blood pressure with single-pill combinations in India (TOPSPIN) – Protocol design and baseline characteristics
Source: Int J Cardiol Cardiovasc Risk Prev. 2024 Oct 24;23:200346. doi: 10.1016/j.ijcrp.2024.200346 (PMC11565424; doi:10.1016/j.ijcrp.2024.200346)
Supplement: Multimedia component 1 [file mmc1.pdf]

Treatment Optimization for blood Pressure with Single-Pill combinations in India (TOPSPIN)

Statistical Analysis Plan

SAP Version 0.2 (Dated: 30 August 2024)
Protocol version-V2.0 02 April 2024
Trial Registration Number: 21IC7054
CTRI registration n: CTRI/2022/04/042106
Clinicaltrials.gov identifier: NCT05683301

Prepared by:
Dimple Kondal & Victoria Cornelius

Table of Contents

1. Administrative information ... 4
1.1 Study Identifiers ... 4
1.2 Revision History ... 4
1.3 Contributors to the Statistical Analysis Plan ... 4
1.3.1 Roles and Responsibilities ... 4
1.3.2 Approvals ... 4
2. Introduction ... 5
3. Study design ... 5
3.1. General design and randomization ... 5
3.2. Treatment regimen ... 6
3.3. Study population ... 6
3.4. Randomization and Blinding ... 6
3.5. Treatment ... 6
3.6. Follow up ... 7
4 Sample size ... 7
5 Trial Endpoints ... 7
6 CONSORT statement ... 8
7 Statistical analysis ... 8
7.1 General Principles ... 8
7.2 Baseline Comparisons ... 8
7.3 Primary Outcome Analysis ... 8
7.4 Secondary outcome analysis ... 9
7.5 Analysis of Missing Outcome Data ... 9
7.6 Sensitivity Analysis and Multiple Imputation ... 9
7.7 Safety Endpoint Analysis ... 9
7.8 Deviations from the SAP ... 9
References ... 10
Appendix – I Template Tables ... 11
Table 1: Demographics and clinical characteristics of the Patients at baseline ... 11
Table 2: Baseline Laboratory Parameters ... 12
Table 3: Clinic Blood pressure measurement ... 12
Table 4: Ambulatory Blood pressure measurement ... 12
Table 5: Follow-up Status ... 14
Table 6: Adjusted mean between-group differences in changes from baseline in Ambulatory Blood Pressure- Primary and Secondary Outcome Analysis ... 15

Table 7. Adjusted mean between-group differences in changes from baseline Clinic Blood Pressure.*-Secondary outcome analysis 16

Figure 1: Clinic Systolic Blood pressure during the trial period 16

Table 8: Rates of Response and Control in Clinic Blood Pressure 17

Table 9. Serious Adverse Events (SAE) or Adverse Events (AE) 18

Supplementary Tables 19

Table S1: Comparison of Baseline Demographic and Clinical Characteristics in Randomized Analysed Patients and without Ambulatory Blood Pressure Monitoring 19

Table S2: Sensitivity analysis: Adjusted mean differences in ambulatory blood pressure between treatment groups 20

Table S2: Sensitivity analysis (Per protocol analysis) Adjusted mean differences in ambulatory blood pressure between treatment groups 20

Table S3: Laboratory Parameters of Patients at Baseline and 6 Months by Study Groups 21

Figure S1: CONSORT 22

Figures S2: Weekly BP variability: Fluctuation of ABPM values over a time 22

Table S4: Unadjusted 6-month ambulatory blood pressure differences (from baseline) by treatment group 23

1. Administrative information

1.1 Study Identifiers

- Protocol version V0.15 28JUL 2021
- Protocol version V1.1 19 APRIL 2022
- Protocol version V2.0 02 April 2024
- Trial Registration Number: 21IC7054

1.2 Revision history

Version	Date	Details
0.1 (draft)	24 July 2024	First version
0.2 (draft)	24 August 2024	Second version

1.3 Contributors to the statistical analysis plan

1.3.1 Roles and Responsibilities

Name and ORCID	Affiliation	Role on Study	SAP contribution
Dimple Kondal 0000-0002-1417-9510	Centre for Chronic Disease Control	Study Statistician	Prepared initial draft
Kavita Singh 0000-0003-4330-666X	Centre for Chronic Disease Control	Co-PI	Reviewed initial draft
Dr. Victoria Cornelius	Imperial College London	Senior Statistician	Reviewed every and final draft
Prof Ambuj Roy	All India Institute of Medical Sciences	PI	Reviewed every draft
Prof D Prabhakaran	Centre for Chronic Disease Control	PI	Reviewed every draft
Prof Neil Poulter	Imperial College London	PI	Reviewed final draft

1.3.2 Approvals

The undersigned have reviewed this plan and approved it as final. We find it to be consistent with the requirements of the protocol as it applies to our respective areas. The SAP is compliant with ICH-E9 principles, and in particular, we confirm that this analysis plan was developed in a completely blinded manner, i.e. without knowledge of the effect of the intervention(s) being assessed.

Approved by	Signature	Date
Prof Ambuj Roy		
Prof D Prabhakaran		
Prof Neil Poulter		
Dr. Victoria Cornelius		

2. Introduction

This document describes the detailed set of statistical analyses to be performed on the data produced by the TOPSPIN trial in conformity with the trial's protocol. The details of the TOPSPIN design can be found in its protocol; here, we report the main characteristics relevant to understanding the chosen data analysis strategy. The document has been finalized before database lock and unblinding.

TOPSPIN is a multi-centre, individual, randomized, single-blind, parallel-group, three-armed trial to compare the efficacy of three single pill combinations (SPCs) of two antihypertensive agents on 24-hour ambulatory systolic blood pressure (ASBP) among individuals with hypertension in India.

The primary objective is to determine which of the three single-pill combinations (SPCs) of two antihypertensive agents is most effective in reducing 24-hour ambulatory systolic blood pressure (ASBP) among individuals with hypertension in India.

The secondary objective(s) are to determine which of the three single pill combinations of two antihypertensive agents is most effective in reducing other measures of BP:

- 24-hour ambulatory diastolic BP (ADBP)
- Daytime and nighttime ambulatory BP and 24-hour BP variability
- Clinic BP at two, four and six months

To determine which of the three single pill combinations of two antihypertensive agents is the most effective in increasing the:

- The proportion of patients who achieve BP control defined as BP: <140/90 mmHg and <130/80 mmHg at any of their clinic visits and maintained at the 6-month clinic visit
- The proportion of patients classified as "responders" defined as those who had a reduction of SBP ≥20 mmHg and DBP ≥10 mmHg at any of their clinic visits and maintained at the 6-month clinic visit.

To determine the effect of three single pill combinations of two antihypertensive agents on:

- Micro- and macro-albuminuria
- Fasting blood glucose
- Fasting lipid profile
- Serum sodium, potassium, urea, creatinine and eGFR
- Adverse events causing trial withdrawal.

To determine whether baseline plasma renin and/or aldosterone predicts any differential BP effects of the three single-pill combinations under investigation.

Safety

We will collect information regarding adverse events causing trial withdrawal, serious adverse events, and unexpected adverse events, which may be related to the implementation of the intervention.

3. Study design

3.1. General design and randomization

This is a randomized, single-blind, multicenter, three-arm trial. The trial was conducted at approximately XX investigational sites in India. Participants were randomized to one of three treatments, as shown in Table 1.

Table 1: Treatment regimens

Treatment Arm	Number of participants	Treatment Period 1: Enrolment – 2 months	Treatment Period 2: 2 months – 6 months (if SBP≥120 mmHg)
1	656	Amlodipine 5 mg and Perindopril 4 mg once daily	Amlodipine 10 mg and Perindopril 8 mg once daily
2	656	Perindopril 4 mg and Indapamide 1.25 mg once daily	Perindopril 8 mg and Indapamide 2.5 mg once daily
3	656	Amlodipine 5 mg and Indapamide 1.5 mg sustained release once daily	Amlodipine 10 mg and Indapamide 1.5 mg sustained release once daily

3.2. Treatment regimen

Patients commenced treatment at the starting doses of the three combinations of "amlodipine + perindopril" or "perindopril + indapamide" or "amlodipine + indapamide (sustained release)". These doses were increased to full doses at the two-month visit, using forced titration if SBP ≥120 mmHg (consistent). If after four months, clinic SBP was >160 mmHg or DBP >100 mmHg, a commonly available beta-blocker (bisoprolol 5mg, which was provided to the trial sites) was added to the trial therapy unless contraindicated, in which case an alternative agent that is not a thiazide or thiazide-like diuretic, ACE inhibitor or CCB such as 12.5 mg of spironolactone or 2 mg of doxazocin was added at the investigator's discretion.

3.3. Study population

The study population consisted of patients referred to secondary and tertiary care clinics/hospitals in India for the management of primary hypertension. In the trial, eligible participants from either tertiary or secondary care centres were enrolled directly, or some primary healthcare settings were invited to participate in the study and enrolled at one of the selected secondary care or tertiary care level hospitals. The details of inclusion and exclusion criteria are provided in the detailed study Protocol version 1.1 19 APRIL 2022.

3.4. Randomization and Blinding

The details are provided in Protocol version 1.1 19 APRIL 2022. In brief, allocation to study groups was performed using variable block randomization in a 1:1:1 allocation ratio and stratified by age (<55 years or ≥ 55 years) and centre.

A web-based central randomization service (centralized electronic clinical data management system-REDCap) at the Public Health Foundation of India was used to randomize patients to one of the three treatment groups. Randomization of study participants was centrally performed by the research coordinating centre in Delhi.

3.5. Treatment

At the randomization visit, participants were dispensed with medication packs containing their study medicines (labelled with the patient's enrolment number) and advised to take one combination tablet daily for the first two months. Given that the packing of drugs was in identical blister packs, patients were blinded to the exact treatment drug being prescribed to them.

After two months, participants were invited for a clinic visit and advised to bring their medication packs. Medication packs (with/without unused tablets) were given to the study coordinator appointed at the site who in turn dispensed a further supply of their allocated study medications (at a higher dose if SBP ≥120 mmHg and as prescribed by the treating physician). Pill counts were carried out by the designated site coordinator at each patient visit (two, four and six-month visits), and these counts will be used to evaluate adherence, and adherence will be defined as ≥80% of doses taken as prescribed. The adherence to medication in the last 24 hours prior to the clinic visit was documented.

3.6. Follow up

Follow-up was at two, four, and six months, and it took place at the outpatient clinic of the study hospital.
At two, four and six-month visits, patients had their sitting blood pressure measured and were asked about compliance and side effects. History of any illness necessitating the patient to see a physician or be hospitalized was also taken. Patients also had their weight checked at the six-month visit.
At the two-month visit, those patients who had not stopped the study medication (because of side effects) and had SBP ≥120 mmHg were force-titrated to receive the higher dose of study medication.
At the four-month visit, if the clinic SBP was >160 mmHg or DBP >100 mmHg, a commonly available beta-blocker (bisoprolol 5 mg once daily) was added to the trial therapy. Bisoprolol was provided to the trial sites. If a beta-blocker was contraindicated, an alternative agent (not a calcium channel blocker or angiotensin converting enzyme inhibitor) such as 12.5 mg of spironolactone or 2mg of doxazosin was added at the physician/investigator's discretion.
At the six-month visit, all participants randomized in the study whilst still on trial treatments underwent phlebotomy, collection of a spot urine sample for detailed assessments and were provided with an ABPM device to wear for 24 hours

4 Sample size

We calculated the sample size to detect a minimum clinically important difference of 3.0 mmHg among the three arms in the 24-hour mean ASBP with the following assumptions: a standard deviation in the 24-hour ASBP to be 15 mmHg, 85% power and a two-sided significance level of 0.0167 for the three comparisons. Based on this, we needed a minimum of 590 participants per arm. Factoring in a 10% dropout rate, we aimed to recruit a total of 1968 participants (656 participants per arm) to achieve 590 evaluable participants per group.

5 Trial Endpoints

The specific trial endpoints, as defined in the protocol, are:

Primary Outcomes

- Differences, between the three trial arms of the mean change in ambulatory systolic blood pressure (ASBP) at six months.

Secondary Outcomes

- Differences, between the three trial arms of the mean change in ambulatory diastolic blood pressure (ADBP) at six months.
- Differences, between the three trial arms of the mean change in Daytime and nighttime ambulatory Systolic and Diastolic BP.
- BP variability measured by ASBP
- Differences between the three trial arms of the mean change in clinic systolic and diastolic blood pressure measured at baseline, 2, 4 and 6 months, respectively.

The difference, between the three trial arms for

- The proportion of patients who achieve BP control defined as BP: <140/90 mmHg and <130/80 mmHg at any of their clinic visits and maintained at the 6-month clinic visit measured at baseline, 2, 4 and 6 months, respectively.
- The proportion of patients classified as "responders" defined as those who had a reduction of SBP ≥20 mmHg and DBP ≥10 mmHg at any of their clinic visits and maintained at the 6-month clinic visit

The difference, tween three trial arms of the proportion or mean change at six months for

- Micro- and macro-albuminuria
  - Microalbuminuria is defined as a urinary albumin-to-creatinine (UACR) level between 30 and 299 mg/g Cr in any follow-up
  - macroalbuminuria is defined as a urinary albumin-to-creatinine (UACR) level of more than 300 mg/g Cr in any follow-up
- Fasting blood glucose
- Fasting lipid profile (Total cholesterol, triglycerides, HDL-c and LDL-c)
- Serum sodium, potassium, urea, uric acid, creatinine and eGFR

6 CONSORT statement

All participants screened for eligibility in this trial will be accounted for, and a CONSORT statement will be prepared (http://www.consort-statement.org), as shown in **Figure 1.** Reasons for early withdrawal will be listed for all participants who prematurely discontinued treatment or left the study. The number of participants who were screened eligible but not randomized will be presented, and the reasons for non-participation (where available) will be recorded.

7 Statistical analysis

7.1 General Principles

Data analyses will be performed by the trial statistician (blind to the randomized allocation) at the Centre for Chronic Disease Control (CCDC), New Delhi. This SAP will be finalized before the final database lock. The statistical analyses will be performed using STATA Version 16.0. The analysis will be performed on the principle of 'intention to treat' unless otherwise specified (i.e., we will compare patients in the groups to which they were originally randomly assigned).

7.2 Baseline comparisons

Baseline descriptive variables of participants will be summarised by treatment arms and overall using suitable measures of central tendencies for continuous data (means and medians), variability (SD and interquartile range (IQR)), and frequencies/percentages for categorical data. No significance testing will be undertaken to compare distributional statistics between arms.

7.3 Primary Outcome Analysis

The primary analysis population will comprise all participants who were randomized and multiple imputation will be used to include participants with missing ASBP measurements at 6 months. For the primary ASBP, a multiple linear regression model will be used to compare the differences in ASBP mean between each of the three randomized arms. The model will include baseline ASBP, the randomization stratification variable age (<55 years or ≥ 55 years), and site; sex as a fixed effect. Model assumptions will be checked through post-estimation plots of residuals, and where the assumptions are not valid, data transformations will be considered. To adjust for multiple hypothesis testing, the p-value < 0.0167 will be considered [Hommel G,

1988; Vickerstaff V, 2019]. The mean difference in ASBP between the groups will be reported with a 95% Confidence interval.
The variables included in the multiple imputation model and details of multiple imputation are in section 7.6. The sensitivity analysis will be performed using a complete case analysis.

An identical analysis will be performed for the secondary outcome of ADBP and the Daytime and nighttime ambulatory Systolic and Diastolic BP.

7.4 Secondary outcome analysis

Clinic systolic and diastolic BPs and other continuous outcomes, which are measured repeatedly over two, four and six months, will be analysed using linear mixed models. The model will include the baseline value of the outcome, the randomization stratification variable age (<55 years or ≥ 55 years), and site; sex as a fixed effect and participant as a random effect; time-by-arm interaction will be included and used to estimate the mean differences between arms at each time point. Binary outcomes, including response to treatment, will be analysed using logistic regression and mixed logistic regression models as appropriate to adjust for randomization stratification variables and a suitable time-to-event model. We do not plan for multiple-comparison adjustments for secondary outcomes.

7.5 Analysis of Missing Outcome Data

The analysis will be conducted according to the intention to treat principle, meaning that participants will be analyzed in the groups to which they will have been randomized regardless of compliance with the protocol. Patients who have discontinued study drugs will be invited to attend scheduled clinic visits. If the participant does not attend study visits, follow-up will be attempted by telephone to obtain information regarding study outcomes and adverse events. The main model will be valid under the missing at random assumption and use multiple imputation to ensure that all randomized participants are included.

7.6 Multiple Imputation

The multiple-imputation analyses will be performed using multiple imputation chained equations for patients with a missing primary endpoint value. We will generate 50 imputed data sets with a maximum of 1000 iterations, with linear imputation for continuous variables and logistic or multinomial regression for categorical variables. Variables that will be included in the imputation model are treatment group, ambulatory systolic and diastolic blood pressures, clinic blood pressure measurements, age, sex, trial site, body mass index (BMI), presence of diabetes or dyslipidemia, duration of hypertension, and pulse rate.

We will perform a sensitivity analysis using complete case analysis and a per-protocol analysis by excluding patients with protocol violations. This sensitivity analysis will cover the primary and secondary outcomes.

7.7 Safety Endpoint Analysis

Information on adverse events (AEs) was collected from several sources: spontaneous reports from participants, clinical examination and observation, and laboratory tests. All AEs will be tabulated by arm and severity for the number of participants with at least one adverse event and the number of adverse events occurring amongst all participants.

7.8 Deviations from the SAP

All deviations from the SAP will be disclosed in the final analysis report. Problems or fundamental issues that become apparent during checking that form part of the statistical analysis will be escalated by the trial statistician to a senior statistician and other appropriate

individuals. Any such action and subsequent decisions will be documented in the final statistical analysis report.

7.9 Examination of Subgroups

We will estimate the effect of the intervention by prespecified sub-groups by including interaction terms in the models as explained above. We will also consider subgroup analyses for the variables listed below:

- Age (<55 and ≥ 55 years)
- Sex (Male; Female)
- BMI (<23; 23-24.99; ≥25 Kg/m^2)
- Baseline Diabetes status (Yes/No)
- Hypertension (Known/Newly Diagnosed)

We will report the credibility of the sub-group analysis using the Instrument for Assessing the Credibility of Effect Modification Analyses (ICEMAN) criteria.

References

1. Schulz KF, Altman DG, Horton JN. CONSORT 2010 statement: updated guidelines for reporting parallel group randomized trials. BMJ 2010; 340:c332
2. Pedroza C, Truong VTT. Estimating relative risks in multicenter studies with a small number of centers — which methods to use? A simulation study. Trials. 2017; 18: 512.
3. 2. Hommel G. A stagewise rejective multiple test procedure based on a modified Bonferroni test. Biometrika. 1988;75(2):383–6
4. 3. Vickerstaff V, Omar RZ, Ambler G. Methods to adjust for multiple comparisons in the analysis and sample size calculation of randomized controlled trials with multiple primary outcomes. BMC Medical Research Methodology. (2019) 19:129

Appendix – I Template Tables

Table 1: Demographics and clinical characteristics of the Patients at baseline

Characteristics	Amlodipine and Perindopril	Amlodipine and Indapamide	Perindopril and Indapamide
Sex-n(%)			
Male			
Female			
Age			
Mean-year			
Distribution-n(%)			
Weight-Kg			
Height-cm			
Body mass index, Kg/m^2			
Blood pressure-mmHg			
Clinic			
Systolic			
Diastolic			
Ambulatory			
Systolic			
24-hr			
Daytime			
Nighttime			
Diastolic			
24-hr			
Daytime			
Nighttime			
Pulse-beats/min			
Self-reported History			
Diabetes mellitus-n(%)			
Dyslipidemia-n(%)			
Current smoking-n(%)			
Current alcohol use-n(%)			
Previous antihypertensive therapy (%)			
Calcium-channel blocker			
Diuretic			
ACE inhibitor			
ARB			
Beta-Blocker			

Values will be presented as mean(SD) or median(p25, p75) or n(%) as appropriate
EGFR= Estimated Glomerular Filtration Rate, LDL= Low Density Lipoprotein, HDL= High Density Lipoprotein

Table 2: Baseline Laboratory parameters

	Amlodipine and Perindopril	Amlodipine and Indapamide	Perindopril and Indapamide
Sodium-mmol/L			
Potassium-mmol/L			
Urea-mmol/L			
Uric acid-mmol/L			
Creatinine-mmol/L			
Estimated eGFR, mL/min/ 1.73m2			
Fasting Glucose-mmol/L			
Total cholesterol-mmol/L			
LDL cholesterol-mmol/L			
HDL cholesterol-mmol/L			
Triglyceride-mmol/L			
Haemoglobin -g/dL			
White Blood Count-10^9/L			

Values will be presented as mean(SD) or median(p25, p75) as appropriate

Table 3: Clinic Blood pressure measurement

	Amlodipine and Perindopril	Amlodipine and Indapamide	Perindopril and Indapamide
Baseline			
Systolic mmHg			
Diastolic mmHg			
Heart rate beats/min			
At 2 month			
Systolic mmHg			
Diastolic mmHg			
Heart rate beats/min			
At 4 month			
Systolic mmHg			
Diastolic mmHg			
Heart rate beats/min			
At 6 month			
Systolic mmHg			
Diastolic mmHg			
Heart rate beats/min			

Values will be presented as mean(SD)

Table 4: Ambulatory Blood pressure measurement

	Amlodipine and Perindopril	Amlodipine and Indapamide	Perindopril and Indapamide

**Mean 24-hour ASBP-mmHg**			
Baseline			
6 months			
Difference in Mean 24-hour ASBP			
**Mean 24-hour ADBP -mmHg**			
Baseline			
6 months			
Difference in Mean 24-hour ADBP			
**Mean Daytime ASBP-mmHg**			
Baseline			
6 months			
Difference in Daytime ASBP			
**Mean Daytime ADBP -mmHg**			
Baseline			
6 months			
Difference in Mean Daytime ADBP			
**Mean Nighttime ASBP -mmHg**			
Baseline			
6 months			
Difference in Mean Nighttime ASBP			
**Mean Nighttime ADBP -mmHg**			
Baseline			
6 months			
Difference in Mean Nighttime ADBP			

Table 5: Follow-up Status

	Amlodipine and Perindopril	Amlodipine and Indapamide	Perindopril and Indapamide
Finished study follow-up			
Lost to follow-up			
Withdrew consent (Subject decision)			
Withdrawn by PI (Doctor decision)			
Withdrawn due to adverse event			
Death			

Table 6: Adjusted mean between-group differences in Ambulatory Blood Pressure- Primary and Secondary Outcome Analysis

Characteristics	Amlodipine and Perindopril Vs Perindopril and Indapamide		Amlodipine and Indapamide vs Perindopril and Indapamide		Amlodipine and Perindopril Vs Amlodipine and Indapamide	
	Mean difference (95% CI)	P Value	Mean difference (95% CI)	P Value	Mean difference (95% CI)	P Value
	mm Hg		mm Hg		mm Hg	
Model 1*						
24-hour						
Systolic						
Diastolic						
12-Hour						
Systolic						
Daytime						
Nighttime						
Diastolic						
Daytime						
Nighttime						
Model 2**						
24-hour						
Systolic						
Diastolic						
12-Hour						
Systolic						
Daytime						
Nighttime						
Diastolic						
Daytime						
Nighttime						

The primary endpoint was the 24-hour ambulatory systolic blood pressure change between baseline and 6 months.

*Model 1 will be adjusted for stratification variables [age (<55 years or ≥55 years) and trial site], sex and the ambulatory systolic blood pressure at baseline.

**Model 2 will be a sensitivity analysis adjusted for the stratification variable [age (<55 years or ≥55 years) and trial site], baseline ambulatory systolic blood pressure, sex, presence of diabetes mellitus or dyslipidemia, body mass index, heart rate, and duration of hypertension.

Table 7. Adjusted mean between-group differences in Clinic Blood Pressure.

*Secondary outcome analysis

Characteristics	Amlodipine and Perindopril Vs Perindopril and Indapamide	Amlodipine and Indapamide vs Perindopril and Indapamide	Amlodipine and Perindopril Vs Amlodipine and Indapamide
	Mean difference (95% CI)	Mean difference (95% CI)	Mean difference (95% CI)
	mm Hg	mm Hg	mm Hg
Systolic			
2 Month			
4 Month			
6 Month			
Diastolic			
2 Month			
4 Month			
6 Month			

The data will be adjusted for randomization stratification variables [age (<55 years or ≥55 years) and trial site], sex and respective baseline blood pressure.

Figure 1: Clinic Systolic Blood pressure during the trial period

Table 8: Rates of Response and Control in Clinic Blood Pressure

	Amlodipine and Perindopril vs Perindopril and Indapamide			Amlodipine and Indapamide vs Perindopril and Indapamide			Amlodipine and Perindopril Vs Amlodipine and Indapamide		
	Amlodipine and Perindopril, n (%)	Perindopril and Indapamide n (%)	Difference 95% (CI)	Amlodipine and Indapamide, n (%)	Perindopril and Indapamide, n(%)	Difference 95% (CI)	Amlodipine and Perindopril, n (%)	Amlodipine and Indapamide, n(%)	Difference 95% (CI)
Response rate*									
2 month									
4 month									
6 month									
The proportion of patients classified as “responders”**									
Control rate^									
2 month									
4 month									
6 month									
Proportion of patients who achieve BP control^^									

*The response rate is the proportion of patients with a reduction in the Clinic SBP ≥20mmHg and Clinic DBP ≥10 mmHg over a given time

**The proportion of patients classified as “responders” defined as those who had a reduction of SBP ≥20 mmHg and DBP ≥10 mmHg at any of their clinic visits and maintained at the 6-month clinic visit.

^ The control rate is the proportion of patients with a Clinic blood pressure of less than 140/90 mm Hg over a given time

^^The proportion of patients who achieve BP control defined as BP: <140/90 mmHg at any of their clinic visits and maintained at the 6-month clinic visit

Table 9. Serious Adverse Events (SAE) or Adverse Events (AE)

SAE or AE	Amlodipine and Perindopril	Amlodipine and Indapamide	Perindopril and Indapamide	All Patients
Any Serious Adverse Events				
Death				
Any adverse event				
Palpitations				
Pedal swelling				
Dry cough				
Dizziness				
Severe headache				
Swollen lips				
Severe difficulty in breathing with wheezing				
Swollen tongue				
Urine frequency (Increased/decreased)				
Incontinence				
Others				

Supplementary Tables

Table S1: Comparison of Baseline Demographic and Clinical Characteristics in Randomized Analysed Patients and without Ambulatory Blood Pressure Monitoring

Characteristics	Patients with 6-month ABPM	Patients without 6-month ABPM	P-value
Sex-n(%)			
Male			
Female			
Age			
Mean-year			
Distribution-n(%)			
Weight, Kg			
Height, cm			
Body mass index, Kg/m^2			
Clinic Blood Pressure-mmHg			
Systolic			
Diastolic			
Ambulatory			
24-hr			
Daytime			
Nighttime			
Diastolic			
24-hr			
Daytime			
Nighttime			
Pulse-beats/min			
Self-reported History			
Diabetes mellitus-n(%)			
Dyslipedemia-n(%)			
Current smoking-n(%)			
Current alcohol use-n(%)			
Previous antihypertensive therapy-n(%)			
Calcium-channel blocker			
Diuretic			
ACE inhibitor			
ARB			
Beta-Blocker			

Table S2: Sensitivity analysis: Adjusted mean differences in ambulatory blood pressure between treatment groups

ABPM	Amlodipine and Perindopril Vs Perindopril and Indapamide		Amlodipine and Indapamide vs Perindopril and Indapamide		Amlodipine and Perindopril Vs Amlodipine and Indapamide	
	Mean difference (95% CI) mm Hg	P Value	Mean difference (95% CI) mm Hg	P Value	Mean difference (95% CI) mm Hg	P Value
24-hour						
Systolic						
Diastolic						
12-Hour						
Systolic						
Daytime						
Nighttime						
Diastolic						
Daytime						
Nighttime						

Adjusted for randomization stratification variables (age <55/>55), sex, site and baseline value

Table S2: Sensitivity analysis (Per protocol analysis) Adjusted mean differences in ambulatory blood pressure between treatment groups

ABPM	Amlodipine and Perindopril Vs Perindopril and Indapamide		Amlodipine and Indapamide vs Perindopril and Indapamide		Amlodipine and Perindopril Vs Amlodipine and Indapamide	
	Mean difference (95% CI) mm Hg	P Value	Mean difference (95% CI) mm Hg	P Value	Mean difference (95% CI) mm Hg	P Value
24-hour						
Systolic						
Diastolic						
12-Hour						
Systolic						
Daytime						
Nighttime						
Diastolic						
Daytime						
Nighttime						

Table S3: Laboratory Parameters of Patients at Baseline and 6 Months by Study Groups

		Amlodipine and Perindopril	Amlodipine and Indapamide	Perindopril and Indapamide	P-value
Urine albumin Creatinine ratio (UACR)	Baseline				
	6 month				
Fasting Blood Sugar, mmol/L	Baseline				
	6 month				
Total Cholesterol, mmol/L	Baseline				
	6 month				
LDL Cholesterol, mmol/L	Baseline				
	6 month				
HDL Cholesterol, mmol/L	Baseline				
	6 month				
Triglyceride, mmol/L	Baseline				
	6 month				
Serum sodium, mmol/L	Baseline				
	6 month				
Serum potassium, mmol/L	Baseline				
	6 month				
Serum Urea, mmol/L	Baseline				
	6 month				
Uric acid, mmol/L	Baseline				
	6 month				
Serum Creatinine, µmol/L	Baseline				
	6 month				
Estimated eGFR, mL/min/1.73m^2	Baseline				
	6 month				

Values will be presented as Mean (SD) or median (p25, p75) as appropriate

Figure S1: CONSORT

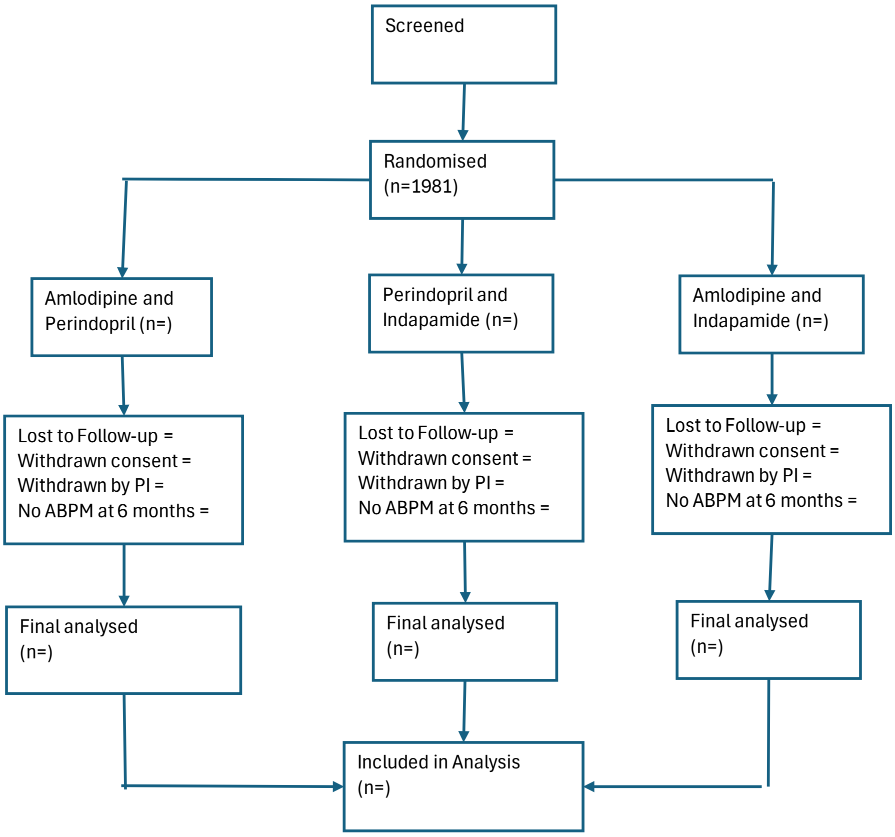

Figures S2: Weekly BP variability: fluctuation of ABPM values over a time

Table S4: Unadjusted 6-month ambulatory blood pressure differences (from baseline) by treatment group

ABPM	Amlodipine and Perindopril Vs Perindopril and Indapamide		Amlodipine and Indapamide Vs Perindopril and Indapamide		Amlodipine and Perindopril Vs Amlodipine and Indapamide	
	Mean difference (95% CI) mm Hg	P Value	Mean difference (95% CI) mm Hg	P Value	Mean difference (95% CI) mm Hg	P Value
24-hour						
Systolic						
Diastolic						
12-Hour						
Systolic						
Daytime						
Nighttime						
Diastolic						
Daytime						
Nighttime						
