# Supplementary material for: Treatment optimisation for blood pressure with single-pill combinations in India (TOPSPIN) – Protocol design and baseline characteristics
Source: Int J Cardiol Cardiovasc Risk Prev. 2024 Oct 24;23:200346. doi: 10.1016/j.ijcrp.2024.200346 (PMC11565424; doi:10.1016/j.ijcrp.2024.200346)
Supplement: Multimedia component 2 [file mmc2.pdf]

Imperial College London

CLINICAL STUDY PROTOCOL

**Study Title:**	**T**reatment **O**ptimisation for blood **P**ressure with **S**ingle-**P**ill combinations in **In**dia<br>**(TOPSPIN)**
**Protocol Number:**	1.0
**Sponsor:**	Imperial College London, London, United Kingdom
**Collaborators:**	Centre for Chronic Disease Control, New Delhi, India<br>All India Institute of Medical Sciences, New Delhi, India
**Clinical Trials Registry India:**	CTRI/2022/04/042106
**Clinicaltrials.gov identifier:**	NCT05683301
**Development Phase:**	Phase 4
**Version no:**	**2.0**
**Protocol Date:**	02 April 2024

This protocol has been developed by the Trial Steering Committee and its contents are the intellectual property of this group. It is an offence to reproduce or use the information and data in this protocol for any purpose other than the trial without prior approval from the project office of the Study

Imperial College London

CONTACT LIST

Principal Investigators

Prof Ambuj Roy, MD, DM (Cardiology), FACC
Department of Cardiology
All India Institute of Medical Sciences,
Ansari Nagar East, New Delhi, Delhi 110029

Tel: +91 11 2659 3861

Prof Dorairaj Prabhakaran, MD, DM (Cardiology), MSc, FRCP, FNASc.
Executive Director,
Centre for Chronic Disease Control (CCDC)
C-1/52, Second Floor,
Safdarjung Development Area,
New Delhi, Delhi 110016

Tel: +91 11 4608 2601

Prof Neil Poulter MBBS, MSc, FRCP, FMedSci
Professor of Preventive Cardiovascular Medicine
Co-Director Imperial Clinical Trials Unit
Imperial Clinical Trials Unit, School of Public Health
Imperial College London, Stadium House, _68 Wood Lane
London W12 7RH

Tel: +44 2075943446

Sponsor

Imperial College London, London, United Kingdom

Collaborators

Centre for Chronic Disease Control, New Delhi
All India Institute of Medical Sciences, New Delhi

Imperial College London

TRIAL COORDINATION

Research Coordinating Centre (RCC) in India

Centre for Chronic Disease Control,
C-1/52, Second Floor,
Safdarjung Development Area,
New Delhi, Delhi-110016

RCC Team in New Delhi, India

Research scientist: Dr. Kavita Singh
Senior research associate:Ms. Raji Devarajan
Data manager:Mr. Mumtaj
Senior biostatistician: Dr. Dimple Kondal

Tel: 011 4608 2601

Imperial Clinical Trials Unit in London, UK

Operations oversight: Dr. Gaia Kiru
Senior statistics oversight: Dr. Victoria Cornelius
Quality assurance oversight: Eloise Britten

ABBREVIATIONS

AE	Adverse event
ACE inhibitor	Angiotensin-converting enzyme inhibitor
ADBP	24-hour ambulatory diastolic blood pressure
ARB	Angiotensin II receptor blocker
ASBP	24-hour ambulatory systolic blood pressure
BP	Blood pressure
CCB	Calcium channel blocker
CRF	Case report form
DBP	Diastolic blood pressure
DSMC	Data safety and monitoring committee
ECDMS	Electronic Clinical Data Management System
eGFR	Estimated glomerular filtration rate
GMP	Good manufacturing practice
ICMJE	International Committee of Medical Journal Editors
ICMR	Indian Council of Medical Research
IEC	Institutional Ethics Committee
IMP	Investigational medicinal product
LVLP	Last visit, last patient
PI	Principal investigator
QA	Quality assurance
SAE	Serious adverse event
SAP	Statistical analysis plan
SBP	Systolic blood pressure
SOP	Standard operating procedure
SSAR	Suspected serious adverse reaction
SUSAR	Suspected unexpected serious adverse reaction
TSC	Trial Steering Committee
UPCR	Urinary protein-to-creatinine ratio

UACR	Urinary albumin-to-creatinine ratio

TABLE OF CONTENTS

CONTACT LIST **2**
TRIAL COORDINATION **3**
ABBREVIATIONS **4**
TABLE OF CONTENTS **5**
TRIAL SUMMARY **8**
1. INTRODUCTION **10**
1.1. Randomised controlled trials in hypertension 10
1.2. Combination therapy in the management of hypertension 12
2. RATIONALE **12**
3. STUDY OBJECTIVES AND ENDPOINTS **13**
3.1. Primary Objective 13
3.2. Secondary Objectives 13
3.3. Primary Endpoint 14
3.4. Secondary Endpoints 14
4. STUDY DESIGN AND METHODS **16**
4.1. Design 16
4.2. Treatment regimens 16
4.3. Study population 17
4.4. Inclusion criteria 17
4.5. Exclusion criteria 17
5. STUDY PROCEDURES AND MEASUREMENTS **18**
5.1. Screening and pre-randomisation evaluation 18
5.2. Randomisation and blinding 19
5.3. Unblinding 20
5.4. Visit Schedule 21
5.5. Treatment 22
5.6. Follow-up 22
5.7. Ancillary and post-trial care 23
5.8. Laboratory Evaluations 23
5.8.1. Biochemistry 23
5.8.2. Urinalysis 24
5.8.3. Pregnancy test 24
5.9. Study schedule 24
6. STUDY INTERVENTION **24**
6.1. Medicinal product 24
6.2 Labelling and Packaging 25

6.3 Storage and Dispensing 25
6.4. Dosage, duration and compliance 25
6.5. Permanent discontinuation of study treatment and withdrawal from study 26
6.5.1. Permanent discontinuation of study treatment 26
6.5.2. Withdrawal from treatment and withdrawal from the study 26
6.5.3. Procedures for withdrawal from study 26
7. PHARMACOVIGILANCE 27
7.1. Adverse Event (AE) 27
7.2. Adverse Event recording 27
8. STATISTICAL ANALYSES 28
8.1 Sample Size and power considerations 28
8.2. Data Analysis 28
8.2.1. Analysis of primary and secondary outcomes 29
8.2.2. Analysis of missing outcome data 30
9. DATA MANAGEMENT 31
9.1. Source Data 31
9.2. Language 31
9.3. Database 31
9.4. Data Collection 32
9.5. Access to data 33
10. REGULATORY, ETHICAL AND LEGAL ISSUES 33
10.1. Declaration of Helsinki 33
10.2. Good Clinical Practice 33
10.3. ICMR Ethical Guidelines 33
10.4. Institutional Ethics Committee Approval 33
10.5. Regulatory Authority Approval 33
10.6. Insurance and Indemnity 34
10.7. Trial registration 34
10.8. Informed consent 34
10.9. Subject Confidentiality 34
11. STUDY MANAGEMENT STRUCTURE 35
11.1. Trial Operations Committee 35
11.2. Trial Steering Committee (TSC) 35
11.3. Data Safety and Monitoring Committee (DSMC) 35
11.4. Monitoring 35
REFERENCES 37
SIGNATURE PAGE 1 (PRINCIPAL Investigator) 40
SIGNATURE PAGE 2 (Co-Principal investigator) 41
SIGNATURE PAGE 3 (Co-Principal investigator) 42
SIGNATURE PAGE 4 (STATISTICIAN) 43

Appendices 44

TRIAL SUMMARY

TITLE: **T**reatment **O**ptimisation for blood **P**ressure with **S**ingle-**P**ill combinations in **In**dia (TOPSPIN)

OBJECTIVES: To compare the efficacy of three single pill combinations (SPCs) of two anti-hypertensive agents on 24 hour ambulatory systolic blood pressure (ASBP) among individuals with hypertension in India.

DESIGN: A multi-centre, individual randomized single-blind, parallel group, three-armed superiority trial.

SAMPLE SIZE: 1968 patients in total, and 656patients per arm.

INCLUSION CRITERIA: Male or female patients aged 30-79 years with a sitting systolic blood pressure (SBP) ≥140 mmHg and <160 mmHg on one antihypertensive agent or sitting clinic SBP ≥150 mmHg and <180 mmHg on no antihypertensive treatment.

EXCLUSION CRITERIA: History or evidence of congestive heart failure, renal impairment, coronary heart disease, cerebrovascular disease, contraindications to the SPCs of investigational product studied, secondary hypertension or other significant illness likely to interfere with the effective conduct of the study, pregnancy or women of childbearing age not taking reliable contraception.

TREATMENT:
Arm 1: SPC of Amlodipine and Perindopril
Arm 2: SPC of Perindopril and Indapamide
Aim 3: SPC of Amlodipine and Indapamide

PRIMARY ENDPOINT:

ASBP at 6 months adjusted for baseline ASBP

SECONDARY ENDPOINTS:

1. 24-hour ambulatory diastolic blood pressure (ADBP) at 6 months adjusted for baseline ADBP
2. Clinic SBP and diastolic blood pressure (DBP) at two, four and six months adjusted for baseline values
3. Daytime and nighttime blood pressure (BP) at six months adjusted for baseline values
4. BP variability measured by ABPM and within-visit clinic BPs

5. Proportion of patients who achieve BP control (primarily defined as clinic BP <140/90 mmHg) at two, four and six months and ABPM measured control (<130/80 mmHg) at six months. In addition to reflect more contemporary guidelines control of clinic BPs will also be evaluated as <130/80 mmHg.
6. Proportion of "responders" (defined as clinic BP reduction ≥20mmHg SBP and ≥10 mmHg DBP) at any of the clinic visits (two, four and six months).
7. Micro- and macro-albuminuria at six months adjusted for baseline values
8. Fasting blood glucose at six months adjusted for baseline values
9. Fasting blood lipid profile at six months adjusted for baseline values
10. Serum sodium, potassium, urea, creatinine and estimated glomerular filtration rate at six months adjusted for baseline values
11. Adverse events causing trial withdrawal

1. INTRODUCTION

Hypertension affects about one quarter of adults and only a minority of treated hypertensive patients get their blood pressures (BPs) controlled to even conservative targets (1-3). Globally, high systolic blood pressure (SBP) is reported as the leading risk factor for morbidity and mortality (2,3). Hypertension is also a leading cause of death and disability in India (4,5) with a prevalence of 30%. Significant variation is noted in hypertension prevalence among rural (28%) and urban (34%) patients in India(6). More importantly hypertension control rates in India are low at 11% and 20% among rural and urban patients respectively (6-8).

Reducing BP to target levels is a major priority in preventing cardiovascular events in patients with hypertension, and typically this requires more than one BP lowering medication in the majority of patients. Single BP lowering drug therapy may help only around 30% of hypertensive patients to achieve the optimal BP control as recommended in recent guidelines (9,10). Current guidelines therefore recommend the use of combination therapy as first-line treatment or early in the management of hypertensive patients with co-morbidities that require prompt BP reduction (9,10,11). The latest European guidelines for treating hypertension in adults recommends initiating treatment for most patients with two anti-hypertensive drugs (10). The combinations recommended in these guidelines are an angiotensin-converting enzyme (ACE) inhibitor or angiotensin II receptor blocker (ARB) with a calcium channel blocker (CCB) or a thiazide/thiazide-like diuretic for management of high blood pressure in people without other comorbidities such as cardiovascular disease or chronic kidney disease. The latest Indian hypertension guidelines recommend combining low doses of two or more drugs in order to achieve the target BP control (11). In addition, initial treatment with two anti-hypertensive medications is suggested for those with BP greater than 20/10 mmHg above the goal pressure by international guidelines (9,10,11).

1.1. Randomised controlled trials in hypertension

Several randomised controlled trials data clearly show that BP reduction is associated with reduced cardiovascular morbidity and mortality (12-16). Furthermore, a meta-analysis of 61 prospective observational studies covering 1 million adults and 12.7

million person-years at risk has substantiated that for every 2mmHg difference in mean systolic blood pressure (SBP), there is a 7% difference in the risk of ischaemic heart disease mortality (17).Randomized controlled trials in hypertension as early as the 1950's showed that patients with malignant hypertension were markedly transformed by the introduction of anti-hypertensive therapy (12). Over the following years (during the 1970's, 1980's and early 1990), trials of severe and moderate hypertension confirmed the protective benefits of lowering BP, with a series of placebo-controlled trials of milder levels of hypertension taking place (12,13). By the end of 1980s, the results of a number of these placebo-controlled trials showed in meta-analysis that a reduction in SBP of 10-12 mmHg and DBP reduction of 6-7 mmHg reduced the incidence of cerebrovascular accident by about 40% (15), which was comparable with the risk reduction reported from prospective observational studies (17). However, the risk of non-fatal myocardial infarction and coronary heart disease death was reduced by about 14% which is less than what was expected from prospective observational data (17).

Following these placebo-controlled trials and the resultant meta-analyses, a series of trials set out to compare the benefits of contemporary drugs over standard therapy. In 2000, the Blood Pressure Lowering Treatment Trialists (BPLTT) Collaboration published a meta-analysis of these trials that included data from approximately 75,000 patients (13). This demonstrated that ARBs were effective in reducing total cardiovascular events, ACE inhibitors and diuretic/beta-blocker-based regimens were more effective than calcium channel blockers in preventing heart failure, and that calcium channel blockers appeared to be more effective in preventing stroke.These analyses were followed by further meta-analyses (14, 15, 16) which showed variable if small differences among the major drug classes in their impact on specific cardiovascular outcomes but in general concluding the degree of BP lowering was of pivotal importance. No robust data were generated regarding optimal treatment thresholds and very few trials addressed the issue of optimal BP targets. This led to conflicting data as to whether or not SBPs should to be lowered to <140mmHg or not and whether this varied according to coexisiting conditions such as diabetes (18-21). More recently the SPRINT trial randomized 9,361 adults aged 50 or above who had SBP≥130 mmHg and at least one other cardiovascular disease risk factor to have their BPs lowered to either <140mmHg or <120mmHg. This study found that in those randomized to the lower target, cardiovascular events were reduced compared with

those randomized to the higher target (22).However, interpretation of these results has been inconsistent (9,10).A systematic review and network meta-analysis of 42 trials, including 144 220 patients comparing targets of blood pressure revealed significant incremental reduction of cardiovascular events and all-cause mortality with lowering systolic blood pressure up to 120-124 mmHg as compared to higher targets. The study thus supported more intensive lowering of blood pressure as in SPRINT trial (23).

1.2. Combination therapy in the management of hypertension

Results from various randomised controlled trials show that the majority of patients with hypertension require at least two BP lowering agents if recommended targets are to be reached (18-20 and 22-28).Consequently, there has been increased emphasis in recent guidelines on the use of combinations of two or three agents on the management of hypertension (9,10).In addition, the superiority of fixed-dose (or single-pill) combinations of antihypertensive agents over monotherapies or two pill combinations has been shown at least in terms of BP-lowering and cost efficacy (28,29,30).

2. RATIONALE

No randomized trial data are available to inform optimal combinations of antihypertensive agents in patients of South Asian origin.

Indeed, no trials comparing optimal BP targets or therapies have been done in India nor have they included enough subjects of South Asian origin to inform drug choice in such patients. Hence current guidelines on drug choices and sequencing in India are based on international guidelines which may or may not be applicable to Indian patients.

In view of this lack of critical information, we propose to compare the efficacy of three different single-pill combinations of antihypertensive therapies (Amlodipine/Perindopril, Perindopril/Indapamide, and Amlodipine/Indapamide)on 24-hour ambulatory BP levels in Indians, as representative of South Asians who constitute one sixth of the world population.

These agents are selected as representative of the three drug classes which are most commonly recommended in combinations in recent hypertension guidelines (9-11).

In summary, the main reasons why this trial is needed are:

a) Hypertension is a big and increasing problem in India with low BP control rates
b) Most hypertensive patients need ≥2 drugs to reach BP targets.
c) There are no randomized controlled trials in a South Asian population comparing two-drug combinations of anti-hypertensive therapies to help physicians make informed choice of BP lowering therapies.

3. STUDY OBJECTIVES AND ENDPOINTS

3.1. Primary Objective

To determine which of three single pill combinations of two anti-hypertensive agents is most effective in reducing 24-hour ambulatory systolic BP (ASBP) in Indian patients with hypertension.

3.2. Secondary Objectives

To determine which of three single pill combinations of two antihypertensive agents is most effective in reducing other measures of BP:

3.2.1. 24-hour ambulatory diastolic BP (ADBP)
3.2.2. Clinic BP
3.2.3. Daytime and nighttime ambulatory BP
3.2.4. BP variability measured by ASBP and within-visit clinic BP

To determine which of the three single pill combinations of two antihypertensive agents is most effective in increasing the:

3.2.5. Proportion of patients who achieve BP control defined as BP: <140/90 mmHg and <130/80mmHg at any of their clinic visits and maintained at the 6-month clinic visit
3.2.6. Proportion of patients classified as “responders” defined as those who had a reduction of SBP ≥20mmHg and DBP ≥10 mmHg at any of their clinic visits and maintained at the 6-month clinic visit

To determine the effect of three single pill combinations of two antihypertensive agents on:

3.2.7. Micro- and macro-albuminuria
3.2.8. Fasting blood glucose
3.2.9. Fasting lipid profile
3.2.10. Serum sodium, potassium, urea, creatinine and eGFR
3.2.11. Adverse events causing trial withdrawal

To determine whether baseline plasma renin and/or aldosterone predicts any differential BP effects of the three single-pill combinations under investigation.

3.3. Primary Endpoint

The primary outcome measure is ASBP at 6 months adjusted for baseline ASBP. This will be calculated as the difference between the mean ASBP at randomization and that at the end of follow up.

Ambulatory blood pressure monitoring (ABPM) is a better reflection of true overall BP and thereby a stronger predictor of adverse cardiovascular events than routine clinic BP readings (30-33).

The primary measurement will be 24-hour ABPM. Patients will wear the ABPM device for a minimum of 24 hours with automatic readings every 30 minutes both in the daytime and at nighttime. Daytime will be defined as 9 AM through 9 PM, and night-time as 12 midnight through 6 AM. Small, medium or large blood pressure cuffs will be utilized as appropriate on the non-dominant arm. ABPM recordings will only be accepted if at least 70% of expected readings are included. ABPM recordings which do not meet this requirement will need to be repeated.

3.4. Secondary Endpoints

3.4.1. Secondary Efficacy Endpoints

1. ADBP at 6 months adjusted for baseline ADBP. The data will be analysed and assembled in a report on the ABPM and a copy of the results will be maintained in the participant's file.

2. Clinic BP: This will be measured by the clinician or trained site investigator using the same calibrated digital device (OMRON BP Machine) at each study visit. The measurements will be taken after each subject has been sitting for at least five minutes. Systolic and diastolic BPs and heart rate will be measured three times at 1-to 2-minute intervals with the average of the last two readings taken as the mean clinic reading. Clinic BP and heart rate will be measured at baseline, 2 months, 4 months and 6 months and the analysis will adjust for baseline values.
3. Daytime and night-time BP: This will be measured using ABPM. Daytime will be defined as 9 AM through 9 PM, and night-time as 12 midnight through to 6 AM.
4. BP variability: Defined as a fluctuation of BP values over a time period, will be measured using 24-hour ABPM measurements and within-visit clinic BP values.
5. Proportion of patients who achieve BP control: This will be measured in clinic at 2, 4 and 6 months. A participant will be defined as having their 'BP controlled' when their BP (average of reading 2 & 3) is recorded to be <140 mmHg SBP and <90 mmHg Diastolic Blood Pressure (DBP) at any of their clinic visits and maintained at the 6-month clinic visit. In addition, 24hr ABPM control (<130/80 mmHg) and clinic BP control defined as <130/80 mmHg will also be evaluated.
6. Proportion of patients classified as "responders": This will be measured in clinic at 2, 4 and 6 months. A participant will be defined as "responders" if they have a reduction of SBP ≥20mmHg and DBP ≥10 mmHg at any of their clinic visits and maintained thereafter.
7. Micro-albuminuria: This will be measured using spot urine analysis for albumin-to-creatinine ratio (UACR) which are preferred to the 24-hour urine collection for quantification of daily proteinuria due to the greater convenience of spot urine sampling. Measurement will be performed at baseline and 6 months.
8. Fasting blood glucose concentration: This will be measured on serum samples collected at baseline and 6 months.
9. Fasting lipid profile: This will be measured on serum samples collected at baseline, and 6 months.
10. Serum sodium, potassium, urea, uric acid, creatinine and eGFR: This will be measured on serum samples collected at baseline and at 6 months, and if further samples are required in keeping with standard clinical practice.

3.4.2. Secondary Safety Endpoints

- Adverse events causing trial withdrawal.
- Serious adverse events (refer to the SAE manual for list of serious adverse events).

4. STUDY DESIGN AND METHODS

This is a randomised, single-blind, multi-center, three-arm superiority trial. The trial will be conducted at approximately 37 investigational sites in India (Attached as Appendix 4). Participants will be randomised to one of three treatments as shown in **Table 1**.

4.1. Design

Randomised single-blind, parallel group, multi-centre, three-arm superiority trial.

4.2. Treatment regimens

Patients will commence treatment at the starting doses of the 3 combinations of “amlodipine+ perindopril” or “perindopril + indapamide”or “amlodipine + indapamide (sustained release)”. These doses will be increased to full doses at the two month’s visit, using forced titration if SBP ≥120 mmHg (consistent) (see **Table 1**).

Table 1: Treatment regimens

**Treatment Arm**	**Number of participants**	**Treatment Period 1: Enrolment – 2 months**	**Treatment Period 2: 2 months – 6 months** (if SBP≥120mmHg)
1	656	Amlodipine 5mg and Perindopril 4 mg once daily	Amlodipine 10mg and Perindopril 8mg once daily
2	656	Perindopril 4 mg and Indapamide 1.25 mg once daily	Perindopril 8mg and Indapamide 2.5 mg once daily
3	656	Amlodipine 5mg and Indapamide 1.5 mg sustained release once daily	Amlodipine 10 mg and Indapamide 1.5 mg sustained release once daily

If after four months, clinic SBP is >160mmHg or DBP >100 mmHg, a commonly available beta-blocker (bisoprolol 5mg, which will be provided to the trial sites) will be added to the trial therapy unless contraindicated, in which case an alternative agent that
is not a thiazide or thiazide-like diuretic, ACE inhibitor or CCB such as 12.5 mg of spironolactone or 2 mg of doxazocin will be added at the investigator's discretion.

4.3. Study population

The study population will consist of patients referred to secondary and tertiary care clinics/hospitals in India for management of primary hypertension. Eligible participants from the primary health care settings may be invited to participate in the study and enrolled at one of the selected secondary care or tertiary care level hospitals in the trial.

4.4. Inclusion criteria

1. Age 30-79 years

   AND

2. Sitting clinic values* of SBP ≥140 mmHg and <160 mmHg on one antihypertensive agent

   OR

3. Sitting clinic values* of SBP ≥150 mmHg and <180 mmHg on no antihypertensive treatment

* Mean of the last 2 of 3 readings.

4.5. Exclusion criteria

1. Congestive heart failure (clinically defined).
2. Patients with a history of intolerance to any of the study medications for example angioedema or dry cough with ACE--inhibitors.
3. Serum creatinine levels greater than 132.6µmol/l (1.5mg/dl)
4. History of coronary heart disease (i.e., chronic stable angina, myocardial infarction or acute coronary syndrome).
5. History of a stroke or other cerebrovascular accident (i.e. transient ischaemic attack or reversible ischaemic neurological deficit).
6. Severe hepatic impairment

7. Treatment with agents causing torsades de pointes
8. Lactation
9. Contraindications to any of the investigational medicinal products as per the SmPc of drugs studied
10. Known or suspected secondary hypertension.
11. Any other concomitant illness, physical or mental impairment that could interfere with the effective conduct of the study.
12. Pregnancy or those of child-bearing age who are not taking reliable contraception.
13. History of Gout.
14. Serum potassium < 3.5mmol/L at screening.

5. STUDY PROCEDURES AND MEASUREMENTS

Hypertensive participants attending or referred to outpatient clinics of participating hospitals at any of approximately 37 sites across India will be recruited for the study. List of sites can be found in **Appendix 4.** Site investigators will screen potential patients with hypertension for trial eligibility. Participant flow chart is found in **Appendix 1**.

5.1. Screening and pre-randomisation evaluation

Eligible patients will be approached regarding potential participation in the study, and the study purpose, procedures, risks, and potential benefits will be explained by the study coordinator and/or principal investigators. Potential patients will be given the opportunity to ask questions. Patients who voluntarily agree to participate in the study will be asked to document their informed consent. Written informed consent must be obtained before the participants undergo screening procedures.

Prior to randomisation, participants will undergo the following screening procedures:

1. Measurement of sitting BP (3 readings) using a calibrated standard sphygmomanometer or calibrated digital device;
2. Collection of blood (a total of 10 ml of blood sample will be drawn) by venesection for measurement of serum sodium, potassium, urea and creatinine and estimation of glomerular filtration rate; and 4 ml of

serum/plasma sample will be stored for future analysis of renin and aldosterone.

3. 24-hour ABPM (for those taking a single drug for hypertension the ABPM will happen whilst still on the pre-trial treatment). ABPM devices will be provided to the research sites for use in the trial, and these devices will be collected at trial closure. The study procedures manual will detail use of the device and including how to view/remove data from the machines.

Following screening tests, participants will be invited to return to the clinic within one week to receive screening results (renal function), return the ABPM device and for randomisation and enrolment if appropriate.

Patients who are not receiving treatment at the time of screening will remain without treatment depending on the judgement of the physician until the commencement of their study medications immediately after randomisation, while those receiving an antihypertensive medication will remain on their previous therapy until they switch to the study drugs after randomisation.

5.2. Randomisation and blinding

Allocation to study groups will be performed using randomization in a 1:1:1 allocation ratio will be blocked and stratified by age (<55 years or ≥ 55 years) and site/centre. A randomisation list will be drawn up centrally for each site and will include enrolment numbers randomly allocated to one of three trial combinations of Amlodipine/Perindopril vs. Perindopril/Indapamide vs. Amlodipine/Indapamide. The randomisation schedule will be provided to the designated study site coordinator in the electronic clinical database management system by the unblinded statistician who is not involved in analysis of the study data. Access to the randomisation schedule during the study will be provided only to the designated site coordinator responsible for packaging and dispensing of the medications. The randomisation schedule and all pharmacy source documents that could link a patient enrolment number to the treatment assignment will remain secure (e.g. in a lock-up facility in the pharmacy with access limited to only unblinded personnel) until notification from the statistician that the database has been locked.

A web-based central randomization service (centralized electronic clinical data management system – (CLINION) used at the Public Health Foundation of India, which is validated for use in clinical trials) will be used to randomise patients to one of the three treatment groups. Randomization of study participants will be centrally performed by the research coordinating center in Delhi. Enrolled patients will take their allocated enrolment numbers to the study site coordinator at the site assigned to dispense the corresponding study medications blister packs labelled with unique patient identification numbers consisting of the centre and randomisation numbers. The label will contain all required information as per GMP (Good Manufacturing Practice) annex 13 including but not limited to, trial reference name/code, dosing instructions, storage requirements, batch number, expiry date, and sponsor contact details. All labelling will be in English. Further details will be included in the study operations manual. After the two-month clinic visit, patients will receive a higher dose of the same medication they were randomized, if their SBP is ≥120 mmHg, and instructed to complete the treatment.
Study coordinator at each site will receive pre-packed study medications labelled with a code consisting of the site id, participant id and treatment arm.

5.3. Unblinding

The study is single-blind since the commercial products do not match each other. However, measures are taken to mask the identity of dispensed medications and include re-packaging the products into identical medication packs with identical labels such that patients and physicians assessing the outcomes are not easily aware of the group assignments. Patients, investigators, and all members of the study team responsible for performing assessments, as well as the medical monitor and sponsor personnel will remain blind to the identity of the treatment from the time of randomisation until time of database lock. An independent statistician based at the central coordinating centre (CCDC) will be the only unblinded personnel for the duration of the study as the statistician will be responsible for the generation of the randomisation schedule and will not be undertaking the analysis or part of the study team. A detailed description of the randomisation schedule will be included in the operations manual.

Emergency un-blinding will only be undertaken when it is mandated for management of the patient. Most often, study drug discontinuation and knowledge of the possible treatment assignments are sufficient to manage the patient. Prior to unblinding, the attending clinician must complete a checklist to document the reason for unblinding and whether alternatives have been explored. Emergency unblinding may be requested by the investigator or his/her designee by contacting the principal investigator at the research coordinating center, Centre for Chronic Disease Control (CCDC) through email or telephone (with subsequent email). The unblinding request must include the participant treatment number, the date, clinical justification, and the investigator’s signature. The request will be kept in the study file. The unblinded treatment allocation will NOT be recorded in the participant case report form (CRF). Emergency un-blinding of the treatment assignment and withdrawal of the participant from the trial will be carried out in exceptional circumstances by the principal investigator.

5.4. Visit Schedule

**Data Collected**	**Visit 0 Screening**	**Visit1 Randomization**	**Visit 2 2 mths***	**Visit 3 4 mths***	**Visit 4 6 mths***
Informed Consent	×	-	-	-	-
Medical and Social History including major changes to smoking, diets and alcohol consumption	×	×	×	×	×
Adverse Events causing trial drug discontinuation, interruption or adjustment<br><br>Also special conditions eg. pregnancy, drug overdoses or intake of IMP by persons other than the patient.	-	-	×	×	×
Serious Adverse Events	-	-	×	×	×
Height	-	×	-	-	-
Weight	-	×	-	-	×
Pulse	×	×	×	×	×
Check for Renal Bruit and Radio Femoral Delay	×	-	-	-	-
24h-Ambulatory BP Measurement⁺	-	×	-	-	×

Clinic BP Measurement	×	×	×	×	×
Phlebotomy(fasting blood glucose and lipid profile, urea, uric acid, creatinine, eGFR, K+, Na+) +/- serum/plasma for storage	×	-	-	-	×
Urine collection for micro-albuminuria and proteinuria	×	-	-	-	×
Study drug dispensing	-	×	×	×	-
Study drug accountability	-	-	×	×	×

*visit window period at 2 month, 4month and 6 months will be +/- 10 days around the actual visit date.
[+] ABPM machine to be returned to the site after 24 hours by the patient, or collection organised by the study team

5.5. Treatment

At the randomisation visit, participants will be dispensed with medication packs containing their study medicines (labelled with the patient's enrolment number) and advised to take one combination tablet each day for the first two months. The packing of drugs will be in identical blister packs so patients will be blinded to the exact treatment drug being prescribed to them.

After two months, participants will be invited for a clinic visit and will be advised to bring their medication packs with them. Medication packs (with/without unused tablets) will be given to the study coordinator appointed at the site who will in turn dispense a further supply of their allocated study medications (at a higher dose if SBP ≥120 mmHg and as prescribed by the treating physician). Tablet counts will be carried out by the designated site coordinator at each patient visit (two, four and six-month visits), and these counts will be used to evaluate adherence, and adherence will be defined as ≥80% of doses taken as prescribed. The adherence to medication in the last 24 hours prior to clinic visit will be documented.

5.6. Follow-up

Follow up will be at two, four and six months, and will take place at the outpatient clinic of the study hospital.

At two, four and six-month visits, patients will have sitting blood pressure measured, and will be asked about compliance and side effects. History of any illness necessitating the patient seeing a physician or being hospitalized will also be taken. Patients will also have their weight checked at the six-month visit.

If at the two months visit, patients have not stopped the study medication because of side effects, their dose of the study medication will be increased to the higher dose if SBP≥120 mmHg.

At the four months visit, if clinic SBP is >160 mmHg or DBP >100 mmHg, a commonly available beta-blocker (bisoprolol 5 mg once daily) will be added to the trial therapy bisoprolol will be provided to the trial sites. Ifa beta-blocker is contraindicated an alternative agent (not a calcium channel blocker or angiotensin converting enzyme inhibitor) such as 12.5 mg of spironolactone or 2mg of doxazosin will be added at the physician/investigator's discretion.

At the six months visit, all participants randomized in the study whilst still on trial treatments will also undergo phlebotomy, collection of a spot urine sample for detailed assessments as per visit schedule 5.4.and be provided with an ABPM device to wear for 24 hours.

5.7. Ancillary and post-trial care

Patients enrolled for this study will be continued on, the allocated study drug until the end of six months and after the ABPM taken at six months. At the end of six months the patient will continue treatment according to the standard care for hypertension management. Once all trial data are analysed, all attending physicians and participating patients will be advised of the optimal drug combination as determined in this trial. Thereafter it will be relatively straightforward for the optimal combination of agents to be prescribed since all the trial drugs are widely available in India.

5.8. Laboratory Evaluations

Laboratory tests will be performed at the local laboratory using standard methods for routine tests.

5.8.1. Biochemistry

Approximately 10ml of venous blood will be obtained at baseline (for screening and prior to randomisation) and at the 6-month clinic visit. Sample collection, storage, transport and preparation will be according to the study hospital protocols, and

analysis will be done at the hospital laboratory. Testing will be performed for fasting blood glucose, glycated hemoglobin (HbA1c), fasting lipid profile, uric acid and serum creatinine. A small amount (4 ml) of participant's serum sample will be stored at the hospital site until the end of the trial, and then shipped to CCDC for future analysis of renin and aldosterone (once funding is available). The study procedure manual will detail the shipment of these samples from the research sites to CCDC.

5.8.2. Urinalysis

Approximately 5ml of freshly voided urine will be obtained at baseline (for screening and prior to randomisation) and at the 6-month clinic visit. Sample collection, storage, transport and preparation will be according to study hospital protocols, and analysis will be at designated laboratory.Testing for proteinuria will use spot urine analysis for urine albumin creatinine ratio (UACR).

5.8.3. Pregnancy test

Pregnancy testing will not be performed routinely as part of this study since patients of childbearing age without any effective contraceptive will be excluded from the study.

5.9. Study schedule

Study set up and obtaining necessary approvals will last six months, recruitment is estimated to last twelve months. Hence last patient, last visit (LPLV) will occur 18 months after the first patient is randomized. Close down will take 6 months after LPLV.

6. STUDY INTERVENTION

The trial will compare the efficacy of three different single-pill combinations of antihypertensive therapies (amlodipine/perindopril, perindopril/indapamide and amlodipine/indapamide) in Indians.

6.1. Medicinal product

The medications used in this study will be manufactured by Serdia Pharmaceutical (India) Pvt Ltd. Investigational medicinal product (IMP) blisters will be packed with patient cards in order to maintain blinding. Patient cards will be labeled with study

label having unique kit number. Labeled patient cards will be assembled in the patient kit having unique kit number. In order to maintain blinding, blinded batch code will be assigned. Clinical packaging and labelling of the study drugs (IMPs) will be carried out by a suitable packaging and labelling vendor.

6.2 Labelling and Packaging

The designated site coordinator in each site will receive pre-packaged study medications from the packaging and labelling vendor. Study medications will be packaged into tamper evident medication packs labelled with a code which is made of the site and patient randomisation numbers. Randomisation codes will be generated centrally at the research coordinating centre in Delhi. Alpha-numeric codes consisting of site ID, participant ID and treatment group ID will be shared with the Serdia team to generate study labels to be used for drug packaging.

6.3 Storage and Dispensing

Study medications will be stored according to standard pharmacy practice, at each of the study sites. Medication packs will be dispensed at the randomisation clinic visit. Study participants will return at two months where the initial medication packs will be returned and new packs of study medication containing the higher dose tablets will be dispensed as prescribed by the treating physician. Site coordinator at each site will be responsible for storage and dispensing of medications.

6.4. Dosage, duration and compliance

Study drug will be dispensed at the randomisation visit and 2-month clinic visit. Treatment will continue until the 6-month clinic visit. Patients will be given standard advice that is consistent with normal clinical practice i.e. to take the medication in the morning/evening and to take the medication with water. Each medication pack will contain 28 tablets and three packs will be dispensed to the participant so medications can last up to 10 weeks, i.e., until the next scheduled clinic visit. Patients will have their follow up visits scheduled at least one week prior to their medication runs out.

Clinical care will not differ from normal clinical practice except for the randomisation and blinding of antihypertensive medications.

To assess compliance with study medications, participants will be asked to bring the medication packs back at each visit. Unused study drug will be returned to the pharmacy, at each study visit and the number of unused or returned drugs will be counted by the designated site coordinator and recorded on a drug accountability log and on the CRF. Optimal compliance being defined as ≥80% of doses taken as prescribed.

6.5. Permanent discontinuation of study treatment and withdrawal from study

Although individual components of these combinations are generally well tolerated with few side effects in day to day practice, any adverse events such as headache, dry cough, leg swelling, and reflex tachycardia – which cause drug discontinuation – will be recorded at every visit and all/any serious adverse events (such as angioneurotic edema which cause hospitalisation) will also be recorded at clinic visits.

6.5.1. Permanent discontinuation of study treatment

Participants may discontinue study medication for the following reasons:

- At the request of the participant.
- Adverse event/ Serious adverse event
- Allergic reaction to a study drug
- If the investigator considers that a participant's health will be compromised due to adverse events or a concomitant illness that develops after entering the study.

6.5.2. Withdrawal from treatment and withdrawal from the study

Withdrawal from the study refers to both complete withdrawal from the study with no further follow up and discontinuation of study treatment and study procedures, or just withdrawal from study treatment. It can occur for the following reasons:

- Participant decision
- Loss to follow-up

6.5.3. Procedures for withdrawal from study

If participants withdraw from the study or treatment, they will not be replaced (10% drop out has been assumed for power calculations). Participants who withdraw from treatment will be invited to continue in the study and will be encouraged to continue

attending study clinics for routine measurements, with a focus on attending the primary 6-month time point. Reasons for withdrawal will be recorded in the CRF and the participants' medical notes.

7. PHARMACOVIGILANCE

7.1. Adverse Events (AEs)

An AE is any untoward medical occurrence in a patient or clinical trial subject who is administered a medicinal product, and which does not necessarily have a causal relationship with this treatment. An AE can therefore be any unfavourable and unintended sign (including an abnormal laboratory finding), symptom, or disease temporally associated with the use of the trial medication, whether or not considered related to the IMP.

A Serious Adverse Event (SAEs)is an AE which:

- Results in death
- Is life-threatening
- Requires hospitalisation, or prolongation of existing inpatients' hospitalisation
- Results in persistent or significant disability or incapacity
- Is a congenital anomaly or birth defect

7.2. Adverse Event recording

Adverse events will be sought by taking a history from each patient during each visit. Adverse events could also be detected when they are volunteered by the patient during visits or between visits. In addition, data will be collected related to special situations such as pregnancy, drug overdoses, intake of study drugs by persons other than the patient. At randomisation, patients will be provided with contact details which they can call or send a text message in the event of adverse events.

All adverse events which cause discontinuation, interruption or adjustment of trial therapy must be recorded on the adverse events CRF. All adverse events should be treated promptly and appropriately. Treatment may include: no action taken with patient under observation, study drug dosage adjusted or temporarily interrupted, study drug permanently discontinued, concomitant medication given, non-drug therapy given and lastly patient hospitalised or the index hospitalisation prolonged.

All adverse events which cause discontinuation, interruption or adjustment of trial therapy should be followed until their resolution, and assessment should be made at each visit or more frequently to monitor any changes in severity, the suspected relationship to the study drug, the necessary interventions to treat it and the outcome.

In order to ensure the safety of the participant, all serious adverse events regardless of suspected causality, which occur after informed consent has been obtained from the patient and until 30 days after the patient has discontinued study drug must be reported to the Research Coordinating Centre based at CCDC within 24 hours of notification of the occurrence. All AE's and SAE are to be reported in compliance to the New Drugs and Clinical Trials Rules, 2019&The Pharmaco-vigilance Programme of India (PvPI)as per local requirements.

8. STATISTICAL ANALYSES

8.1 Sample Size and power considerations

In order to detect a minimum clinically important difference of 3.0 mmHg between arms in the 24-hour mean ASBP assuming a standard deviation in 24-hour ASBP of 15 mmHg with 85% power and adjusting for three comparisons using a two-sided significance level of 0.0167, we will need a minimum of 590 participants per arm. Factoring in a 10% dropout rate we aim to recruit a total of 1968 participants (656 participants per arm), in order to achieve a total of 590 evaluable participants per group.

8.2. Data Analysis

There are three formal comparisons of interest:

- Amlodipine/Perindopril vs. Perindopril/Indapamide
- Amlodipine/Indapamide vs. Perindopril/Indapamide
- Amlodipine/Perindopril vs. Amlodipine/Indapamide

A separate statistical analysis plan will be prepared and finalised prior to database lock.

8.2.1. Analysis of primary and secondary outcomes

The data analysis will be performed by the trial statistician who will be blind to randomised allocation for the primary and secondary outcome analysis. The trial will be reported using the Consolidated Standards of Reporting Trials (CONSORT) checklist.

Baseline descriptive variables of participants will be summarised by treatment arms and overall using suitable measures of central tendencies for continuous data (means and medians), variability (SD and interquartile range (IQR)), and frequencies/percentages for categorical data. No significance testing will be undertaken to compare distributional statistics between arms.

The primary analysis population will comprise all participants who were randomized and multiple imputation will be used to include participants with missing ASBP measurements at 6 months. For the primary analysis of ASBP a linear mixed model will be used to compare the differences in ASBP mean between each of the three randomised arms. The model will include baseline ASBP and the randomisation stratification variable age (<55 years or ≥ 55 years), as fixed effects. Recruitment site will be included as a random effect unless there are fewer than expected sites or another reason to model site as a fixed effect [34]. An unstructured covariance matrix will be used, and the model will be fitted using restricted maximum likelihood (REML). Model assumptions will be checked through post estimation plots of residuals and where the assumptions are not valid data transformations will be considered. The Hommel method will be used to adjust for multiple hypothesis testing to control for FWER [35] as this method is shown to provide highest disjunctive power [36]. The variables included in the multiple imputation model will be pre-specified in the detailed statistical analysis plan (SAP) and include primary model covariates. Sensitivity analysis will be performed to examine the impact of missing data under a missing not at random assumption using controlled imputation, we will also perform a complete case analysis.
An identical analysis will be performed for the secondary outcome ADBP.

Clinic systolic and diastolic BPs and other continuous outcomes which are measured repeatedly over two, four and six months will be analysed using linear mixed models

that include the baseline value of the outcome, randomisation stratification variables as fixed effects and participant as a random effect, time-by-arm interaction will be included and used to estimate the mean differences between arms at each timepoint Binary outcomes including response to treatment will be analysed using logistic regression and mixed logistic regression models as appropriate to adjust for randomisation stratification variables as well as using a suitable time-to-event model. The Stata (TX, USA) statistical software version 16.0 will be used for data analysis.

Information on adverse events (AEs) will be collected from several sources: spontaneous reports from participants; clinical examination and observation; clinical and laboratory tests. Adverse events provided by the clinical investigators will be coded based on terms included in the Medical Dictionary for Regulatory Activities (MedDRA). MedDRA will be used as a guide for coding of adverse events. The cumulative hazard function will be used to examine rates of withdrawals by arm due to any AE. All AEs will be tabulated by arm and severity for the number of participants with at least one adverse event, and the number of adverse events occurring amongst all participants. We will also calculate adjusted ORs/IRRs and their 95% CIs for binary and count AE outcomes where frequency permits, using logistic regression and Zero-Inflated Poisson model or negative Binomial model, adjusting for stratification variables, and site either as a random effect or fixed effect (following the decision made for the primary analysis).

8.2.2. Analysis of missing outcome data

The analysis will be conducted according to the intention to treat principle, meaning that participants will be analysed in the groups to which they will have been randomised regardless of compliance with the protocol. Patients who have discontinued study drug will be invited to attend scheduled clinic visits. If the participant does not attend study visits, follow up will be attempted by telephone to obtain information regarding study outcomes and adverse events. The main model will be valid under the missing at random assumption and use multiple imputation to ensure that all randomized participants are included. A sensitivity analysis will be undertaken to explore departures from MAR (missingness at random) assumption using controlled multiple imputation.

A detailed statistical analysis plan will be written and finalised prior to the first extract of data from the database (38).

9. DATA MANAGEMENT

9.1. Source Data

Data will be collected directly from the participants during recruitment, randomisation and follow up visits using CRFs administered by study investigators.

A CRF (source notes) will be completed in English for each randomised patient at each study visit. Study personnel and the principal investigator at each site will be responsible for evaluating the CRFs for accuracy and completeness before entering into the online electronic clinical data management system. Once entered, the data manager will review the data for discrepancies and missing data. Source data verification will be performed by the trial monitors according to section 11.4 below. The site will then be informed to make any required corrections and/or additions and the CRF's stored until all analyses are completed.

The principal investigators and the study site staff will be required to respond to the data queries and confirm and correct the data. A printed copy of the CRF will be maintained in the participant's file. Confidentiality of participant's data will be maintained in accordance with national laws with the informed consent form containing a statement describing the extent to which confidentiality of the participant will be maintained.

9.2. Language

CRFs will be in English. Generic names for concomitant medications should be recorded in the CRF wherever possible. All written material to be used by participants will use vocabulary that is clearly understood and be in the language appropriate for the study site.

9.3. Database

Study personnel at the participating sites will be responsible for completing CRFs through remotely accessing a centralized electronic clinical data management system – CRDR platform (CLINION) used at the Public Health Foundation of India, which is

validated for use in clinical trials). Appropriate training will be provided to the clinic site teams for entering data into the CLINION – electronic data capture system by the trial manager.

CCDC will be serving as the Research Coordinating Centre and will have access to all the study data. Individual sites will have access to their own centres' data and will be provided site-specific data summaries by CCDC's data management team, upon request. Source documentation supporting the trial information will be stored at the investigator site and made available for trial related monitoring, audits, institutional ethics committee (IEC) review and regulatory inspections when required. The investigator will retain all study records and files in accordance with applicable regulatory requirements. The data management plan will outline the procedures and guidelines to ensure data quality is adhered to. The collaboration in this study especially between the CCDC, New Delhi, India and Imperial College London, United Kingdom will help build and develop capacity in CCDC as they learn from the wealth of experience of Imperial College London led by Professor Neil Poulter (co-PI). Scientific aspects of the study will be co-supervised by Professor Neil Poulter (co-PI) of Imperial College London, UK, and Professor D. Prabhakaran, CCDC, who have vast experience on trials of hypertension.

Patient information will be stored in a high security computer system and kept strictly confidential. Subject confidentiality will further be ensured by utilizing a subject identification code number to correspond to treatment data on the computerized files. Only the Data Management and Monitoring team will be aware of the unblinded data until the trial is completed or a recommendation is made to terminate the trial.

9.4. Data Collection

Details of procedures for CRF completion will be provided in a study manual. The patients' demographic data, medical and social history (including date of birth, gender, occupation, history of hypertension, diabetes mellitus, alcohol consumption and smoking habits) and findings on examination will be recorded on the online CRF in the electronic clinical data management system. The height and weight of the patients will be measured during enrolment by a trained site coordinator with the patients standing

and wearing light clothing with no shoes. Body mass index will be calculated as weight in kilograms divided by the square of height in meters (kg/m^2).

9.5. Access to data

CCDC will have access to all the study data. Individual sites will have access to their own centres' data. Each site personnel who is responsible for entering and reviewing participant data on the Electronic Clinical Data Management System will be provided access with a secure password. Sites will be provided with site-specific data summaries by CCDC, upon request.

10. REGULATORY, ETHICAL AND LEGAL ISSUES

10.1. Declaration of Helsinki

The study investigators will ensure that this study is conducted in full conformity with the 7th revision of the 1964 Declaration of Helsinki.

10.2. Good Clinical Practice

The study will be conducted in accordance with the latest guidelines laid down by the International Conference on Harmonisation for Good Clinical Practice (ICH-GCP).

10.3. ICMR Ethical Guidelines

The study will be conducted in accordance with Indian Council of Medical Research's (ICMR) National Ethical Guidelines for Biomedical and Health Research Involving HumanParticipants-2017.

10.4. Institutional Ethics Committee Approval

All investigators will obtain ethical approval from their institutional ethics committees. Imperial College Research Ethics Committee (ICREC)have also provided a favourable opinion for the trial.

10.5. Regulatory Authority Approval

The study will be performed in compliance with that country's regulatory requirements. Since the IMP is already available in India, Drug Controller General of India (DCGI)

approval is not required. Approval from the Health Ministry's Screening Committee (HMSC) will be obtained prior to the start of the study.

10.6. Insurance and Indemnity

The sponsor, Imperial College London, UK, has purchased insurance coverage for this study in accordance with the laws and regulations of the country.

10.7. Trial registration

The study will be registered on Clinical Trial Registry India and Clinicaltrials.gov in accordance with requirements of the International Committee of Medical Journal Editors (ICMJE) regulations.

10.8. Informed consent

The Investigator or the person designated by the Investigator should fully inform the patient of all the important aspects of the clinical trial including approval of the trial by the ethics committee. Prior to the participation of the patient in the study, the informed consent form should be signed and personally dated by the patient and by the site investigator/delegate.

Illiterate participants should place their thumbprint in the dedicated space on the consent form, and the form signed by the legally acceptable representative/witness as well as the site investigator/delegate.

The informed consent forms will be checked by the trial Monitor from CCDC at the on-site visits.

10.9. Subject Confidentiality

Study data sent to the Electronic Clinical Data Management System at the Research Coordinating Centre at CCDC will be securely stored. Source documents pertaining to the study maintained in each participating site will be stored securely by the site coordinator in accordance with the ethical requirements and local procedures. Informed consent will be obtained from the participants to collect, transmit and store pseudo anonymised personal information.

Any data collected as a result of the trial will be available for inspection by CCDC, Imperial College London, the site ethics committees and regulatory authorities as required by law. Servier will have access to tabulated/aggregated data only as well as trial reports and documents.

11. STUDY MANAGEMENT STRUCTURE

11.1. Trial Operations Committee

A Trial Operations Committee consisting of members of CCDC and AIIMS will meet by telephone conference on approximately a weekly basis (or less frequently if deemed appropriate) to address the operational matters of the study, and to implement the decisions of the Trial Steering Committee.

11.2. Trial Steering Committee (TSC)

The Trial Steering Committee will consist of Ambuj Roy (co-PI),Dorairaj Prabhakaran (PI), Neil Poulter (co-PI), Anushka Patel (Chair), Nikhil Tandon, and Denis Xavier.This committee will be chaired by Professor Anushka Patel. The committee will meet half yearly to review study progress. Any changes to the composition of the TSC will be documented appropriately.

11.3. Data Safety and Monitoring Committee (DSMC)

An external and independent Data Safety and Monitoring Committee (DSMC) will monitor the trial for safety and efficacy. The DSMC will review the data for safety at 6 months, 12 months and at the trial end or as determined by the DSMC. The team will comprise of three members including a biostatistician. Members of the DSMC include: Dr. PP Mohanan (Cardiologist with clinical trial experience), Dr. Nitish Naik (Cardiologist with clinical trial experience) and Dr. RM Pandey (Biostatistician).Any changes to the composition of the DSMC will be documented appropriately.

11.4. Monitoring

In addition to the site initiation visit and close-out monitoring visits, site monitoring visits will be conducted according to the risk-based monitoring plan. This will likely entail at least one to two site monitoring visits for each of the centers by the trial monitor to ensure that all the sites adhere to the protocol and trial operating

procedures. Centralized (remote) statistical monitoring will also be performed at regular intervals (quarterly) to identify any data outliers or inliers. Monthly teleconferences will be conducted between the RCC, and the site PI and study coordinator where feedback will be received from all the sites regarding trial conduct. Quality Assurance (QA)and Operations Oversight will also be supplied by Imperial Clinical Trials Unit (ICTU).

In the case that external factors (eg. the Covid-19 pandemic) do not allow on-site monitoring visits at any point during the course of the trial, alternative procedures may be implemented (eg. remote SDV or self-compliance checklists) to ensure ongoing quality and safety.

Imperial College London

REFERENCES

1. Poulter NR, Prabhakaran D, Caulfield M. Hypertension. Lancet. 2015;386(9995):801-12.
2. Roth GA, Johnson C, Abajobir A, et al. Global, Regional, and National Burden of Cardiovascular Diseases for 10 Causes, 1990 to 2015. Journal of the American College of Cardiology 2017; 70: 1–25.
3. Mills KT, Bundy JD, Kelly TN, et al. Global Disparities of Hypertension Prevalence and Control: A Systematic Analysis of Population-Based Studies From 90 Countries. Circulation 2016; 134: 441–50.
4. Gupta R, Xavier D. Hypertension: The most important non communicable disease risk factor in India. Indian Heart J. 2018;70(4):565-572.
5. Gupta R, Gaur K, S Ram CV. Emerging trends in hypertension epidemiology in India. J Hum Hypertens 2019; 33: 575–87.
6. Anchala R, Kannuri NK, Pant H, et al. Hypertension in India. A systematic review and meta-analysis of prevalence, awareness, and control of hypertension. Journal of hypertension 2014; 32: 1170–77.
7. Roy A, Praveen PA, Amarchand R, et al. Changes in hypertension prevalence, awareness, treatment and control rates over 20 years in National Capital Region of India: results from a repeat cross-sectional study. BMJ Open. 2017;7(7):e015639.
8. Prenissl J, Manne-Goehler J, Jaacks LM, et al. Hypertension screening, awareness, treatment, and control in India. A nationally representative cross-sectional study among individuals aged 15 to 49 years. PLoS medicine 2019; 16: e1002801. doi:10.1371/journal.pmed.1002801.
9. Whelton PK, Carey RM, Aronow WS, et al. 2017 ACC/AHA/AAPA/ABC/ACPM/AGS/APhA/ASH/ASPC/NMA/PCNA Guideline for the Prevention, Detection, Evaluation, and Management of High Blood Pressure in Adults: Executive Summary: A Report of the American College of Cardiology/American Heart Association Task Force on Clinical Practice Guidelines. Hypertension. 2017;71(6):1269-1324.
10. Williams B, Mancia G, Spiering W, et al. 2018 ESC/ESH Guidelines for the management of arterial hypertension: The Task Force for the management of arterial hypertension of the European Society of Cardiology and the European Society of Hypertension: The Task Force for the management of arterial hypertension of the European Society of Cardiology and the European Society of Hypertension. *J Hypertens* 2018; **36**(10): 1953-2041.
11. Standard treatment guidelines. Hypertension: Screening, Diagnosis, Assessment, and Management of Primary Hypertension. May 2016. Ministry of Health & Family Welfare, India. http://clinicalestablishments.gov.in/En/1068-standard-treatment-guidelines.aspx (accessed Nov 03, 2019).
12. Collins R, Peto R, MacMahon S, Hebert P, Fiebach NH, Eberlein KA, et al. Blood pressure, stroke, and coronary heart disease. Part 2, Short-term reductions in blood pressure: overview of randomised drug trials in their epidemiological context. Lancet. 1990;335(8693):827-38.
13. Neal B, MacMahon S, Chapman N, Blood Pressure Lowering Treatment Trialists C. Effects of ACE inhibitors, calcium antagonists, and other blood-pressure-lowering drugs: results of prospectively designed overviews of randomised trials. Blood Pressure Lowering Treatment Trialists' Collaboration. Lancet. 2000;356(9246):1955-64.
14. Law MR, Morris JK, Wald NJ. Use of blood pressure lowering drugs in the prevention of cardiovascular disease: meta-analysis of 147 randomised trials in the context of expectations from prospective epidemiological studies. BMJ. 2009;338:b1665.
15. Thomopoulos C, Parati G, Zanchetti A. Effects of blood pressure lowering on outcome incidence in hypertension: 7. Effects of more vs. less intensive blood pressure lowering and different achieved blood pressure levels - updated overview and meta-analyses of randomized trials. J Hypertens. 2016 Apr;34(4):613-22.
16. Ettehad D, Emdin CA, Kiran A, Rodgers A et al. Blood pressure lowering for prevention of cardiovascular disease and death: a systematic review and meta-analysis.Lancet (London, England) 2016; 387: 957–67. doi:10.1016/S0140-6736(15)01225-8.

17. Lewington S, Clarke R, Qizilbash N, Peto R, Collins R, Prospective Studies C. Age-specific relevance of usual blood pressure to vascular mortality: a meta-analysis of individual data for one million adults in 61 prospective studies. Lancet. 2002;360(9349):1903-13.
18. Brunstrom M, Carlberg B. Effect of antihypertensive treatment at different blood pressure levels in patients with diabetes mellitus: systematic review and meta-analyses. BMJ 2016;352:i717.
19. Xie X, Atkins E et al. Effects of intensive blood pressure lowering on cardiovascular and renal outcomes: updated systematic review and meta-analysis. Lancet 2016;387:435-443.
20. The ACCORD Study Group. Effects of intensive blood pressure control in Type 2 Diabetes Mellitus. NEJM 2010;362:1575-1585.
21. Appel LJ, Wright JT, Jr., Greene T, Agodoa LY, Astor BC, Bakris GL, et al. Intensive blood-pressure control in hypertensive chronic kidney disease. The New England journal of medicine. 2010;363(10):918-29.
22. SPRINT Research Group, Wright JT Jr, Williamson JD, Whelton PK, et al. A Randomized Trial of Intensive versus Standard Blood-Pressure Control. N Engl J Med. 2015 Nov 26;373(22):2103-16.
23. Bundy JD, Li C, Stuchlik P, et al. Systolic Blood Pressure Reduction and Risk of Cardiovascular Disease and Mortality: A Systematic Review and Network Meta-analysis. *JAMA Cardiol.* 2017;2(7):775–781.
24. Dahlof B, Sever PS, Poulter NR, et al for the ASCOT investigators. Prevention of cardiovascular events with an antihypertensive regimen of amlodipine adding perindopril as required versus atenolol adding bendroflumethiazide as required, in the Anglo-Scandinavian Cardiac outcomes Trial – Blood Pressure Lowering Arm (ASCOT-BPLA): a multicentrerandomised controlled trial. Lancet 2005;366:895-906.
25. MacDonald TM, Williams B, Webb DJ, et al. Combination Therapy Is Superior to Sequential Monotherapy for the Initial Treatment of Hypertension. A Double-Blind Randomized Controlled Trial. Journal of the American Heart Association 2017; 6. doi:10.1161/JAHA.117.006986.
26. Mourad J-J, Waeber B, Zannad F, Laville M, Duru G, Andréjak M. Comparison of different therapeutic strategies in hypertension. A low-dose combination of perindopril/indapamide versus a sequential monotherapy or a stepped-care approach. Journal of hypertension 2004; 22: 2379–86.
27. Webster R, Salam A, de Silva HA, et al. Fixed Low-Dose Triple Combination Antihypertensive Medication vs Usual Care for Blood Pressure Control in Patients With Mild to Moderate Hypertension in Sri Lanka: A Randomized Clinical Trial. JAMA. 2018;320(6):566-579.
28. Gupta AK, Arshad S, Poulter NR. Compliance, safety, and effectiveness of fixed-dose combinations of antihypertensive agents: a meta-analysis. Hypertension (Dallas, Tex. :1979) 2010; 55: 399–407.
29. Salam A, Kanukula R, Atkins E, et al. Efficacy and safety of dual combination therapy of blood pressure-lowering drugs as initial treatment for hypertension. A systematic review and meta-analysis of randomized controlled trials. Journal of hypertension 2019; 37: 1768–74.
30. O'Brien E, Parati G, Stergiou G, Asmar R, Beilin L, Bilo G, et al. European Society of Hypertension position paper on ambulatory blood pressure monitoring. Journal of hypertension. 2013;31(9):1731-68.
31. Hansen TW, Jeppesen J, Rasmussen S, Ibsen H, Torp-Pedersen C. Ambulatory blood pressure monitoring and risk of cardiovascular disease: a population based study.Am J Hypertens. 2006; *19*:243–250.
32. Clement DL, De Buyzere ML, De Bacquer DA, et al. Office versus Ambulatory Pressure Study Investigators. Prognostic value of ambulatory blood-pressure recordings in patients with treated hypertension.N Engl J Med. 2003; *348*:2407–2415
33. Niiranen TJ, Mäki J, Puukka P, Karanko H, Jula AM. Office, home, and ambulatory blood pressures as predictors of cardiovascular risk.Hypertension. 2014; *64*:281–286.
34. Pedroza C, Truong VTT. Estimating relative risks in multicenter studies with a small number of centers — which methods to use? A simulation study. Trials. 2017; 18: 512.
35. Hommel G. A stagewise rejective multiple test procedure based on a modified Bonferroni test. Biometrika. 1988;75(2):383–6.

36. Vickerstaff V, Omar RZ, Ambler G. Methods to adjust for multiple comparisons in the analysis and sample size calculation of randomised controlled trials with multiple primary outcomes. BMC Medical Research Methodology. (2019) 19:129
37. Hewitt CE, Torgerson DJ, Jeremy NVM. Is there another way to take account of noncompliance in randomized controlled trials? CMAJ. 2006 Aug 15; 175(4): 347.
38. Cro S, Morris TP, Kenward MG, Carpenter JR: Sensitivity analysis for clinical trials with missing continuous outcome data using controlled multiple imputation: A practical guide. Stat Med 2020, 39(21):2815-2842.

SIGNATURE PAGE 1 (PRINCIPAL INVESTIGATOR)

The signature below constitutes approval of this protocol by the signatory and provides the necessary assurances that this study will be conducted according to all stipulations of the protocol including all statements regarding confidentiality.

Study Title: **T**reatment **O**ptimisation for blood **P**ressure with **S**ingle-**P**ill combinations in **In**dia (TOPSPIN)

Protocol Version: 2.0

Signed:

Investigator Name:Prof. Dorairaj Prabhakaran

Study Site Name: Centre for Chronic Disease Control, New Delhi

Date:

Imperial College London

SIGNATURE PAGE 2 (CO-PRINCIPAL INVESTIGATOR)

The signatures below constitute approval of this protocol by the signatory.

Study Title: **T**reatment **O**ptimisation for blood **P**ressure with **S**ingle-**P**ill combinations in **In**dia (TOPSPIN)

Protocol Version: 2.0

Signed: ____

Investigator Name: _Prof. Neil Poulter

Study Site Name: Imperial College London, UK

Date: ______________________

SIGNATURE PAGE 3 (CO-PRINCIPAL INVESTIGATOR)

The signatures below constitute approval of this protocol by the signatory.

Study Title: **T**reatment **O**ptimisation for blood **P**ressurewith **S**ingle-**P**ill combinations in **In**dia (TOPSPIN)

Protocol Version: 2.0

Signed:

Investigator Name: Prof. Ambuj Roy

Study Site Name: All India Institute of Medical Sciences, New Delhi

Date:

Imperial College London

SIGNATURE PAGE 4 (STATISTICIAN)

The signatures below constitute approval of this protocol by the signatory.

Study Title: **T**reatment **O**ptimisation for blood **P**ressure with **S**ingle-**P**ill combinations in **In**dia (TOPSPIN)

Protocol Version: 2.0

Signed: ____

Investigator Name:___________

Study Site Name: Imperial College London, London, UK___

Date:

/APPENDICES

Appendix 1: Participant Flow Chart

Appendix 2: Publications Policy

Appendix 3: Authorship Guidelines

Appendix 4: List of Participating investigation sites

Appendix 1: Participant Flow Chart

Screen male and female patients aged 30-79. Eligibility with a sitting SBP ≥140mmHg and <160 mmHg on one antihypertensive agent, or sitting SBP ≥ 150 mmHg and <180 mmHg on no antihypertensive treatment

↓

Complete Screening/Enrolment Form

↓

Obtain signed informed consent

↓

Phlebotomy: Obtain results for Fasting blood glucose, HbA1c, lipid profile, uric acid, serum creatinine

Urine collection for urine albumin creatinine ratio (UACR)

↓

Clinical exam and social history, check pulse and renal bruit and radio femoral delay

Patient is eligible (Proceedfurther) → **Randomize the patient (Blinded)**

Patient noteligible → Record reasons on screening log**Visit 1**

Arm 1:

Amlodipine + Perindopril

Arm 2:

Perindopril + Indapamide

Arm 3:

Amlodipine + Indapamide

Study Schedule Visits

Visit 2	Visit 3	Visit 4 (Final)
2 months	4 months	6 months

Appendix 2: Publications policy

Publication policy and authorship will be coordinated through the Publications Committeethat reports directly to the Steering Committee of which it is a sub-committee. Guidelines for the Publications Committee are as follows:

Professor Ambuj Roy will chair the Publications Committee. Other members of the committee are Prof Neil Poulter, Professor Dorairaj Prabhakaran, Dr Kavita Singh and Dr Gaia Kiru.

The overall purpose of the Publications Committee is to:
- Ensure the timely writing and publication of high-quality manuscripts from trial data;
- Solicit, review and prioritize analysis plans and manuscripts from the trial database;
- Prioritize authorship of manuscripts / abstracts / posters arising from the trial database in a fair and just manner;
- Review manuscripts / abstracts / posters arising from the trial database.

Appendix 3:Authorship Guidelines

Primary Publications: It is anticipated that at least two primary publications should result if the trial is successful. The first (methods) will describe the rationale, design and methods of the trial. The second (results) will describe the outcome of the trial. The main paper will be led by Prof Ambuj Roy, Prof Prabhakaran and Prof Neil Poulter. All other key collaborators will be co-authors. All actively recruiting centres will be listed in an appendix under the name of the Principal Investigator and his / her organisational affiliation.

Secondary Publications: These may arise from sub-studies or from suggestions from participants who wish to analyse the trial database in areas of personal interest. In such instances the Publications Committee will be responsible for ensuring that first and last authors are the people that have developed the project idea and done the writing and also for ensuring that all listed authors meet conventionally accepted authorship credit guidelines. Coordinating center team may be included as authors at the discretion of the Publications Committee.

Appendix 4: List of Participating investigation sites

No	Investigator Name	Name of Institution	Address of Institution
1	Dr. Amit Malviya	North Eastern Indira Gandhi Regional Institute of Medical Sciences	Mawdiangdiang, East Khasi Hills ,Shillong 793018, Meghalaya
2	Dr. Kiran Aithal (Site PI), Dr. Satish G Patil (Co-I)	SDM College of Medical Sciences & Hospital	Shri Dharmasthala Manjunatheshwara University, Manjushree Nagar, Sattur, Dharwad, Karnataka
3	Dr. Bishav Mohan	Dayanand Medical College and Hospital	Tagore Nagar, Civil Lines, Ludhiana, Punjab
4	Dr. Shantanu Sengupta	Sengupta Hospital & Research Institute	Ravinagar Square , Nagpur 440033 , Maharashtra , India
5	Dr. Nagendra Boopathy	Sri Ramchandra Institute of Higher Education and Research (SRIHER)	1, Ramachandra Nagar, Porur, Chennai
6	Dr. P.V.Raghava Sarma	Lalitha Super Specialities Hospital Pvt Ltd	Kothapet, Guntur, Andhra Pradesh
7	Dr. Vinod K Abichandani	Rudraksh Hospital	Rudraksha Speciality Hospital, 1st Floor Raj Mandir Complex, off Bareja fly over, Bareja, Ahmedabad.
8	Dr. Neil Bardoloi	Apollo Excelcare Hospital	NH-37, near Ganesh Mandir, Paschim Boragaon, Guwahati, Assam 781033
9	Dr. G. Justin Paul	Madras Medical College	EVR Salai, Park Town, Chennai-600002
10	Dr. Jabir Abdullakutty	Lisie Hospital	Lisie Hospital, P.B. No:3053, Kochi, Kerala-682018
11	Dr. Surender Deora, Dr. Jaykaran Charan (co-PI) - not involved	All India Institute of Medical Sciences, Jodhpur	Basni - II Phase
12	Dr. Ambuj Roy	All India Institute of Medical Sciences, New Delhi	Ansari Nagar, New Delhi- 110029

13	Dr. Jaigopal P B	Lakshmi Hospital	Chittur Road, Palakkad - 678013 Mysuru
14	Dr. Sunil Kumar	JSS Hospital, Mysuru	HOD, Department of Cardiology, JSS Medical College Hospital,
15	Dr. B K Gupta	S P Medical College, Bikaner	Department of Medicine, S.P. Medical College & A.G. of Hospitals Bikaner, Rajasthan
16	Dr. Bhupinder Singh	AIIMS, Bhatinda	Department of Cardiology All India Institute of Medical Sciences Bhatinda, Punjab
17	Dr. Balsubramaiam Yellapantula	Shalinitai Meghe Hospital and Research Center, Nagpur	Dept of Medicine, Shalinitai Meghe Hospital and Research Center Higna Road, Wandongari Nagpur, Maharashtra
18	Dr. H C Kalita	Assam Medical College, Dibrugarh	Dept. of Cardiology, Assam Medical College, Dibrugarh, Assam
19	Dr. Sharan Badiger	BLDE (Deemed to be University), Vijayapura	Dept of Medicine, BLDE (Deemed to be University) Shri B. M. Patil Medical College, Hospital & Research Centre Vijayapur, Karnataka
20	Dr. Rakesh Sahay	Osmania General Hospital, Hyderabad	Department of Endocrinology, Osmania General Hospital, Hyderabad
21	Dr. Lovleen Bhatia	Govt Medical College, Patiala	Dept. of Medicine, Government Medical College, Patiala, Punjab
22	Dr. Sudhir Varma	Sadbhavna Medical and Heart Institute, Patiala	Sadbhavna Medical & Heart Institute Opposite State College of Education, Patiala, Punjab
23	Dr. Hemant Thacker	Bhatia Hospital, Mumbai	Bhatia Hospital, G-1 Block [Ground Floor), Tukaram favji Road, Tardeo, Mumbai, Maharashtra
24	Dr. Aman Khanna	Aman Hospital & Research Center, Vadodara	Aman Hospital and Research Center 15, Shashwat, Opp ESI Hospital, Gotri Road, Vadodara, Gujarat
25	Dr. D Sreeramulu	Kurnool Medical College, Kurnool	Dept. of General Medicine, Kurnool Medical College, Kurnool, Andhra Pradesh

26	Dr. Partha Saradhi SV	Apollo DRDO Hospitals, Hyderabad	Dept. of Nephrology, Apollo DRDO Hospitals, Kanchanbagh, Hyderabad, Telangana
27	Dr. Anitha Kolukula	Apollo Hospitals, Visakhapatnam	Apollo Hospitals, Health City, Visakhapatnam -530040
28	Dr. R Prahalad	Apollo Institute of Medical Sciences, Hyderabad	Dept of General Medicine, Apollo Institute of Medical Sciences & Research Hyderabad.
29	Dr. Debomallya Bhuyan	Nazareth Hospital, Shillong	Dept. of Medicine, Nazareth Hospital, Shillong, Meghalaya
30	Dr. O R Kumaran	Apollo Hospitals, Madurai	Dept of Internal Medicine, Apollo Hospital, Madurai, Tamil Nadu
31	Dr. Yusuf A Kumble	Indiana Hospital & Heart Institute, Mangalore	Dept of Cardiology, Indiana Hospital & Heart Institute Pumpwell, Kankanady Post Mangalore, Karnataka
32	Dr. Prashant Kr Sahoo	Apollo Hospitals, Bhubaneshwar	Department of Cardiology Apollo Hospitals Enterprise Limited Plot No-251, Sainik School Road, Unit-15 Bhubaneswar, Odisha
33	Dr. D Shailendra	Mediciti Institute of Medical Sciences, Hyderabad	Dept. Pharmacology, MediCiti Institute of Medical Sciences, Hyderabad, Telangana
34	Dr. L Sreenivasa Murthy	Lifecare Hospital & Research Centre, Bangalore	Lifecare Hospital and Research Centre Sahakaranagara, Bangalore, Karnataka
35	Dr. Suvarna Patil	BKL Walawalkar Rural Medical College and Hospital, Devran	Dept. of Medicine, BKL Walawalkar Rural Medical College and Hospital, Devran, Maharashtra
36	Dr. Vinit Shah	Apollo Hospitals, Ahmedabad	Dept. of Cardiology, Apollo Ahmedabad, Bhat Gidc Estate, Gandhinagar, Gujarat
37	Dr. Saptarshi Bhattacharya	Indraprastha Apollo Hospitals, New Delhi	Dept. of Endocrinology, Indraprastha Apollo Hospitals, Sarita Vihar, New Delhi
